# 3D Artificial Array Interface Engineering Enabling Dendrite-Free Stable Zn Metal Anode

**DOI:** 10.1007/s40820-022-01007-z

**Published:** 2023-01-17

**Authors:** Jianbin Ruan, Dingtao Ma, Kefeng Ouyang, Sicheng Shen, Ming Yang, Yanyi Wang, Jinlai Zhao, Hongwei Mi, Peixin Zhang

**Affiliations:** 1 College of Chemistry and Environmental Engineering, Shenzhen University, Shenzhen, 518060 People’s Republic of China; 2 Institute of Microscale Optoelectronics, Shenzhen University, Shenzhen, 518060 People’s Republic of China; 3 College of of Materials Science and Engineering, Shenzhen University, Shenzhen, 518060 People’s Republic of China; 4 Guangdong Flexible Wearable Energy and Tools Engineering Technology Research Center, Shenzhen, 518060 People’s Republic of China

**Keywords:** Aqueous Zn-ion batteries, Volume stress, 3D artificial array interface, Controllable deposition, Zn metal anode

## Abstract

The ripple effect induced by uncontrollable Zn deposition is considered as the Achilles heel for developing high-performance aqueous Zn-ion batteries. For this problem, this work reports a design concept of 3D artificial array interface engineering to achieve volume stress elimination, preferred orientation growth and dendrite-free stable Zn metal anode. The mechanism of MXene array interface on modulating the growth kinetics and deposition behavior of Zn atoms were firstly disclosed on the multi-scale level, including the in-situ optical microscopy and transient simulation at the mesoscopic scale, in-situ Raman spectroscopy and in-situ X-ray diffraction at the microscopic scale, as well as density functional theory calculation at the atomic scale. As indicated by the electrochemical performance tests, such engineered electrode exhibits the comprehensive enhancements not only in the resistance of corrosion and hydrogen evolution, but also the rate capability and cyclic stability. High-rate performance (20 mA cm^−2^) and durable cycle lifespan (1350 h at 0.5 mA cm^−2^, 1500 h at 1 mA cm^−2^ and 800 h at 5 mA cm^−2^) can be realized. Moreover, the improvement of rate capability (214.1 mAh g^−1^ obtained at 10 A g^−1^) and cyclic stability also can be demonstrated in the case of 3D MXene array@Zn/VO_2_ battery. Beyond the previous 2D closed interface engineering, this research offers a unique 3D open array interface engineering to stabilize Zn metal anode, the controllable Zn deposition mechanism revealed is also expected to deepen the fundamental of rechargeable batteries including but not limited to aqueous Zn metal batteries.

**Supplementary Information:**

The online version contains supplementary material available at 10.1007/s40820-022-01007-z.

## Highlights

- The design concept of 3D artificial array interface engineering was proposed to achieve eliminated volume stress, preferred orientation growth and dendrite-free Zn metal anode.
- The mechanism of MXene array interface on modulating the growth kinetics and deposition behavior of Zn atoms were firstly uncovered at a multiscale level.
- The engineered electrode exhibits comprehensive enhancements in the resistance of corrosion and hydrogen evolution reaction, as well as the rate capability and cyclic stability.
- 3D MXene array@Zn/VO_2_ batteries with enhanced rate performance (214.1 mAh g^−1^ at 10 A g^−1^) and cycle lifespan can be demonstrated.

**Supplementary Information:**

The online version contains supplementary material available at 10.1007/s40820-022-01007-z.

## Graphical abstract

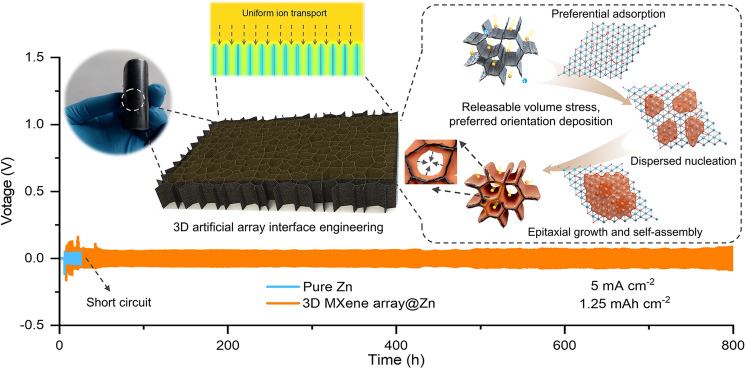

## Introduction

Low-cost, save and environmental-friendly aqueous Zn-ion batteries (AZIBs) have been considered as a promising candidate for energy storage applications [1–4]. Unfortunately, for Zn metal anode, uneven deposition would disrupt the distribution equilibrium of electric field near the anode surface, thus accelerating the formation of Zn dendrite (Fig. S1a) [5, 6]. Notably, the as-formed high Young’s modulus of zinc dendrite would further pierce the separator and making the batteries short circuit [7]. Thus, how to realize the controllable Zn deposition at the electrolyte/anode interface has become the critical question for developing durable AZIBs.

According to the previous studies, the effective strategies to control the Zn deposition can be mainly divided into the following two categories. One is to introduce two-dimensional (2D) zincophilic interface to homogenize the adsorption site and achieve the uniform deposition [8]. However, such ordered results usually depend on the stability and flatness of interface, yet the competitive and even dominant growth of high electrochemical activity and low thermodynamic stability-crystal planes such as Zn(101) and Zn(100), are still unable to effectively avoid [9]. Take a flat and dense interfacial layer for example (Fig. S1b), on one hand, such strategy is usually hard to accommodate large-capacity deposition owing to the less space under the interface [10]. Besides, during the reversible plating/stripping process, the accumulation of volumetric stress would destroy the interface layer, and the collapsed interface would finally induce random deposition and dendrite formation [11, 12].

Excepting that, another way is to introduce a lattice-matched interface layer as inactive substrate to guide the preferred orientation deposition [13, 14]. Typically, such ordered deposition is mainly realized by regulating the growth kinetics of crystal plane at the atomic level. For zinc metal, it has been demonstrated that Zn(002) exhibits the lowest electrochemical activity and highest thermodynamic stability than that of other crystal planes such as Zn(100) and Zn(101), owing to its higher atomic packing density and homogeneous interfacial charge density distribution [15]. Therefore, the preferred orientation deposition along Zn(002) should be expected to essentially inhibit side reactions [16, 17]. However, apart from the graphene-based substrate materials, other potential substrates still need to be developed [7, 9, 15]. In particularly, Ti_3_C_2_T_x_ MXene has been indicated as another promising candidate owing to its low lattice mismatch rate (about 10%) with zinc metal in theory [18, 19]. However, in previous works about MXene-modified zinc metal anodes, most of them only focused on its role in homogenizing electric field, while ignored its underlying effect on influencing the texture growth of zinc metal [10, 20–22]. Let the modulation mechanism of Ti_3_C_2_T_x_ MXene substrate on the growth kinetics and deposition behavior of Zn atoms is still not distinct. More importantly, beyond the common 2D closed interface engineering, how to construct a three-dimensional (3D) open interface with abundant adsorption sites, enough space to resist the volume stress, as well as facilitate the interfacial transport kinetics should be highly suggested yet still be a challenge.

Inspired by the mentioned-above idea, take the 2D MXene nanosheets as skeleton, this work presents a 3D artificial array interface engineering to achieve dendrite-free stable Zn metal anode (3D MXene array@Zn). In this design concept, the vertically aligned interface is able to homogenize the Zn ion flux and facilitate the fast ion/electron transport, while open channels assembled by zincophilic MXene skeleton can not only provide abundant adsorption sites and release the volume stress effect, but also guide the Zn(002)-preferred orientation Zn deposition. Notably, the mechanism of MXene array interface on regulating the growth kinetics and deposition behavior of zinc atoms were studied via theoretical calculation and multiple in-situ technologies. Benefited from the synergistic effect of such 3D artificial array interface, it was demonstrated that the 3D MXene array@Zn electrode can deliver an ultra-stable cycling performance over 1500 h at 1 mA cm^−2^. Even at the higher current density of 5 mA cm^−2^ with a fixed areal capacity of 1.25 mAh cm^−2^, it can also steadily work over 800 h. Moreover, both of rate capability and cyclic stability of Zn/VO_2_ batteries also can be promoted via adopting such rationally designed electrode.

## Experimental Section

### Materials Synthesis

#### ***Few-Layers Ti***_***3***_***C***_***2***_***T***_***x***_*** MXene Nanosheets***

Ti_3_C_2_T_x_ nanosheets were synthesized by a typical top-down method etching from Ti_3_AlC_2_ ceramic. The etchant was prepared by added 2 g LiF to 25 mL HCl (37%) and then stirred for 30 min to obtain the homogeneous etchant (or directly use 49% HF as etchant). After that, 1 g Ti_3_AlC_2_ ceramic (supplied by Xinxi technology.) was slowly added to the etchant in several times. Notably, the above steps were carried out under ice bath condition, to avoid the violently reaction between Ti_3_AlC_2_ ceramic and the etchant. Next, the container was sealed and stirred at 40 °C for 24 h. Subsequently, the acquired dispersion was repeated washed by de-ionized water and centrifuged at a speed of 3500 rpm for 5 min, until the pH value reached about 6. The few-layers Ti_3_C_2_T_x_ nanosheets were exfoliated by hand shaped the obtained Ti_3_C_2_T_x_ suspension violently for 5 min, and then centrifuged at 3500 rpm for 1 h. The obtained supernatant was then treated by freeze-dried to produce the few-layers Ti_3_C_2_T_x_ nanosheets.

#### *3D MXene array@Zn Anode*

100 mg few-layers Ti_3_C_2_T_x_ nanosheets powder was dispersed into 1 mL sodium alginate solution (1%) and stirred for 40 min to form the homogenous slurry. Subsequently, coated the slurry onto Zn foil (100 µm thickness), and the Zn foil was immediately put onto a cold finger to freeze and form the vertically array structure. Finally, using a freeze dryer to dehydrate the as-prepared 3D MXene array@Zn anode. Stamping it into a disc with a diameter of 14 mm before use, and the mass of MXene array is about 0.66 mg cm^−2^.

#### ***VO***_***2***_*** Cathode***

The details of VO_2_ synthesis can be refer to Na Li et al.’s work [23]. Typically, 1.092 g V_2_O_5_, 2.268 g H_2_C_2_O_4_·2H_2_O and 0.04 g CTAB were mixed and added to 70 mL de-ionized water. Then, the mixed solution was stirred for 2 h, following by transferred to a 100 mL auto-clave Teflon liner and maintained at 180 °C for 48 h. The blue precipitate can be obtained by centrifuged the resulted solution. After washed with de-ionized water and absolute ethanol for several times and dried at 80 °C for 12 h, the VO_2_ was finally synthesized. Stamping it into a disc with a diameter of 14 mm before use.

### Materials Characterization

The crystal structure and elemental valence were investigated by powder X-ray diffractometer (XRD, Empyrean) and X-ray photoelectron spectroscopy (XPS, Thermo Fisher, K-Alpha). The morphology was characterized by transmission electron microscopy (TEM, JEM-2100) and field emission scanning electron microscope (FESEM, JSM-7800F). The deposition behavior of Zn was observed through the optical microscope (Motic-MRL50). Raman spectra was acquired by using a LabRAM HR Evolution (HORIBA Jobin Yvon, France) Raman microscope.

In-situ XRD measurement of 3D MXene array@Zn electrode during the continues Zn planting process for 8 h was carried out at a constant current density of 0.5 mA cm^−2^. In-situ optical observation was used to monitor the changes of cross-section morphology of Zn anode during continuous deposition. The test was carried out at a constant current density of 10 mA cm^−2^ for 20 min. In-situ Raman spectrum of 3D MXene array@Zn anode during a discharge/charge cycle for 30 min was carried out at a constant current density of 0.5 mA cm^−2^. And the wavelength of incident light is 532 nm.

### Electrochemical Measurements

The electrochemical performance of symmetric cell and full cell are both test in the form of encapsulated CR2032-type coin cells with 140 µL 3 M Zn(CF_3_SO_3_)_2_ aqueous electrolyte, glass fiber separator (Whatman GF/D). The galvanostatic charge–discharge curve and cycling performance were tested via the LAND CT2001A battery test system. The Zn/Zn symmetric cell were cycled at the area current density at 0.5, 1, 5 and 20 mA cm^−1^ with specific areal capacity of 0.5, 1, 1.25, and 10 mAh cm^−2^. And the 3D MXene array@Zn/VO_2_ batteries were cycled at the current density of 0.5 and 5 A g^−1^, and the pure Zn anode (about 100 μm) was used as a control group. the loading of VO_2_ is about 1–2 mg cm^−2^. Cyclic voltammetry (CV), liner cyclic voltammetry (LSV) and electrochemical impedance spectroscopy (EIS) were measured by the Solartron electrochemical station (1470E). The frequency of EIS spectrum ranges from 0.01 to 100,000 Hz. The Tafel plot test used a three-electrode system (Working electrode: Zn, Counter electrode: Pt, Reference electrode: Ag/AgCl, electrolyte: 1 M NaSO_4_ solution) with a scan rate of 1 mV s^−1^. And the HER plot test uses the same three-electrode system, except that the electrolyte was changed to 3 M Zn(CF_3_SO_3_)_2_.

### Computational Details

The present first principle DFT calculations are performed by Vienna Ab initio Simulation Package (VASP) with the projector augmented wave (PAW) method [24, 25]. The exchange-functional is treated using the generalized gradient approximation (GGA) of Perdew-Burke-Ernzerhof (PBE) functional [26]. The energy cutoff for the plane wave basis expansion was set to 450 eV and the force on each atom less than 0.02 eV Å^−1^ was set for convergence criterion of geometry relaxation. The Brillouin zone integration is performed using 3 × 3 × 1. The self-consistent calculations apply a convergence energy threshold of 10^–5^ eV. The adsorption energy was calculated according to formula (1):

1 $${\mathrm{E}}_{\mathrm{ads}}={E}_{\mathrm{total}}-{E}_{\mathrm{base}}-{E}_{\mathrm{Zn}}$$

where *E*_total_ is the total energy of the Zn adsorbed system, *E*_base_ and *E*_Zn_ are the energies of the pure base structure and the isolated Zn atom, respectively.

### Simulation Details

Simulation in this work were performed by COMSOL multiphysics based on a nonlinear phase-field model by finite element method using a two-dimensional model [27]. The physics model used was “Electrodeposition Tertiary Nernst–Planck”. The Zn-ion flux was given by the Nernst–Planck equation. The boundary conditions for the anode and cathode were given by the Butler–Volmer equation. This study focuses on initial Zn ion flux (including Zn ion distribution and electrolyte current density distribution) and it was found that the Zn ion flux changed very little along with the increasing time of Zn deposition. The simulation results were collected after 0.1 s of simulation time. The potential of electroplating was set to − 25 mV *vs*. Zn/Zn^2+^. Initial Zn-ion concentration was set to 3.0 M. The diffusion coefficient of Zn ion in the electrolyte was set to 3.3 × 10^–10^ m^2^ s^−1^. The average current density through the cell was set as *i*_0_ = 5 A m^−2^. For the pure Zn electrode, a triangle with a side length of 10 microns is set at an interval of 15 microns to simulate the protrusion on the zinc foil. For the 3D MXene array@Zn, a 60 microns high vertical diaphragm analog MXene array is set at an interval of 15 microns.

## Results and Discussion

### Synthesis and Characterizations of 3D MXene Array@Zn Anode

**Fig. 1:**
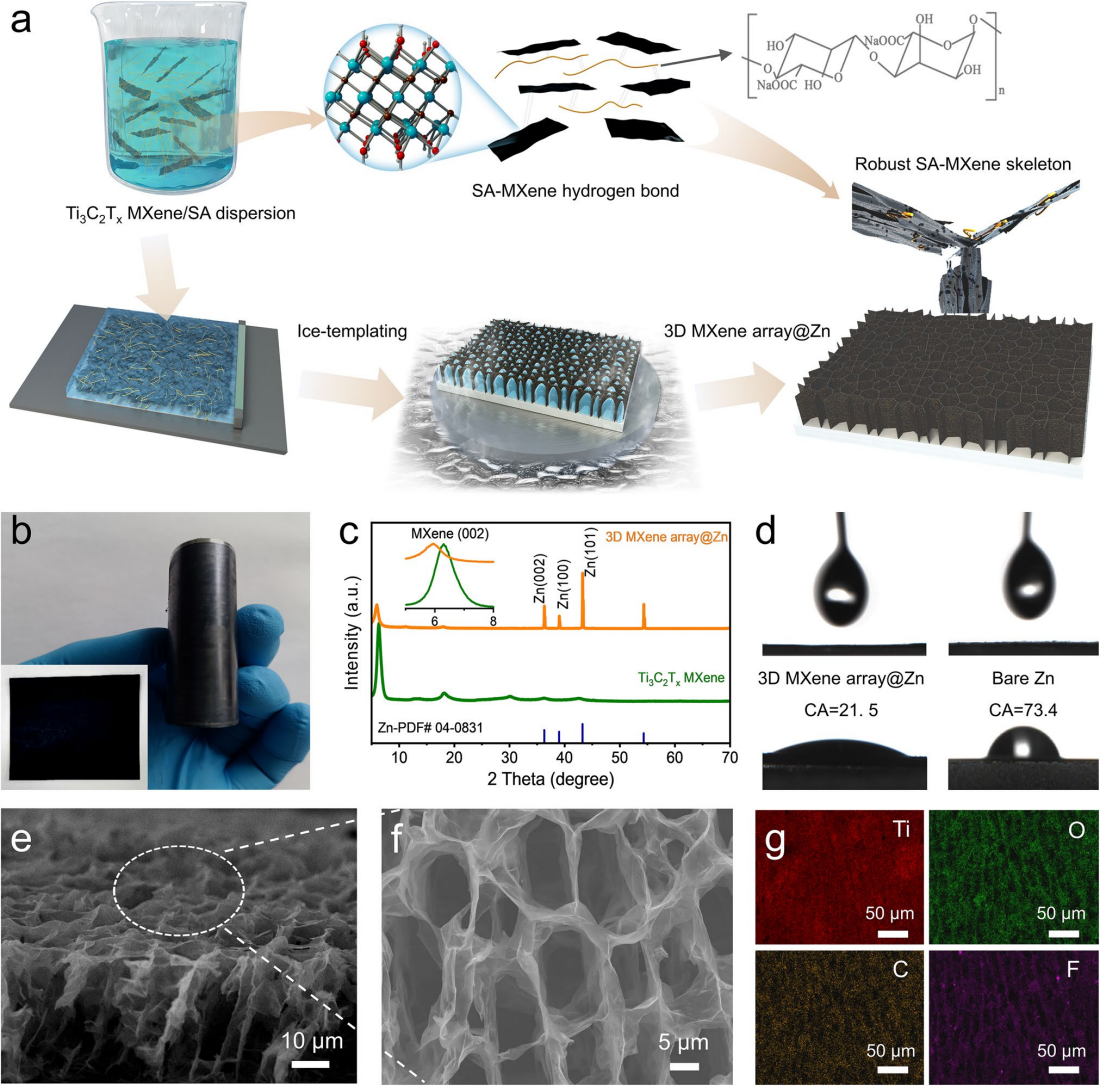
**a** Schematic illustration of the fabrication process of 3D MXene array@Zn. **b** Optical image of 3D MXene array@Zn foil. **c** XRD patterns of the Ti_3_C_2_T_x_ MXene and 3D MXene array@Zn. **d** The contact angle of 3 M Zn(CF_3_SO_3_)_2_ against 3D MXene array@Zn and pure Zn foil. **e** Side-view and **f** top-view FESEM images of 3D MXene array@Zn foil, and **g** corresponding EDS mapping

3D MXene array@Zn foil was fabricated via the ice-template method, as illustrated in Fig. 1a. Among them, Ti_3_C_2_T_x_ MXene is the conductive and zincophilic skeleton, while the introduction of hydroxyl groups-enriched sodium alginate (SA) binder is expected to strengthen the stability of array interface via the formation of SA-MXene hydrogen bond. Then, the digital image (Fig. 1b) shows that a black coating layer was uniformly covered on the pure Zn foil. No cracks can be found even after bending the modified Zn foil, indicating a good adhesion between MXene array interface and zinc foil. XRD patterns (Fig. 1c) confirm the well match of Ti_3_C_2_T_x_ MXene with the previous reports, the successful etching of Ti_3_AlC_2_ can be further proved by the result of XPS spectra (Fig. S2) [28]. Notably, the intensity of diffraction peak of MXene (002) would significantly decrease yet shift toward a lower angel in the engineered Zn foil. On one hand, the decreased intensity of MXene (002) should be mainly caused by the special structure of MXene array@Zn [10, 29, 30]. Since the array structure would change the diffraction direction of the incident X-ray and reduces the X-ray received by the detector, resulting in the decreased intensity of MXene (002) [31]. On the other hand, the interaction between SA and MXene enlarges the layer spacing of MXene, causing the diffraction peak of MXene (002) shift toward a lower angel [32, 33]. Attributed to the abundant hydrophilic terminal groups on the surface of MXene skeleton, the contact angel refers to the 3 M Zn(CF_3_SO_3_)_2_ electrolyte significantly decreases to 21.5° for 3D MXene array@Zn (Fig. 1d), implying it an enhanced electrolyte permeability. Then, the morphology of Ti_3_C_2_T_x_ MXene and 3D MXene array@Zn foil were characterized. The Ti_3_C_2_T_x_ MXene possess a micron-sized flake morphology (Fig. S3) after etching. And AFM characterization confirms it with an ultrathin thickness of ~ 5 nm (Fig. S4). Subsequently, the side view (Fig. 1e) and top view (Fig. 1f) FESEM images of 3D MXene array@Zn foil demonstrate the construction of vertically aligned array on the Zn foil. Corresponding EDS mapping (Fig. 1g) also confirms the uniform distribution of Ti, C, O and F elements along the certain orientation. Moreover, the thickness of array interface can be easily regulated by adjusting the height of scraper (Fig. S5). Unlike the 2D closed interface layer, such 3D artificial array interface with low tortuosity and open channel can be expected to not only facilitate the efficient transport kinetics, but also release the volume stress during reversible Zn plating/stripping process [34].

### Zn Plating/Stripping Performance of 3D MXene Array@Zn Anode

**Fig. 2:**
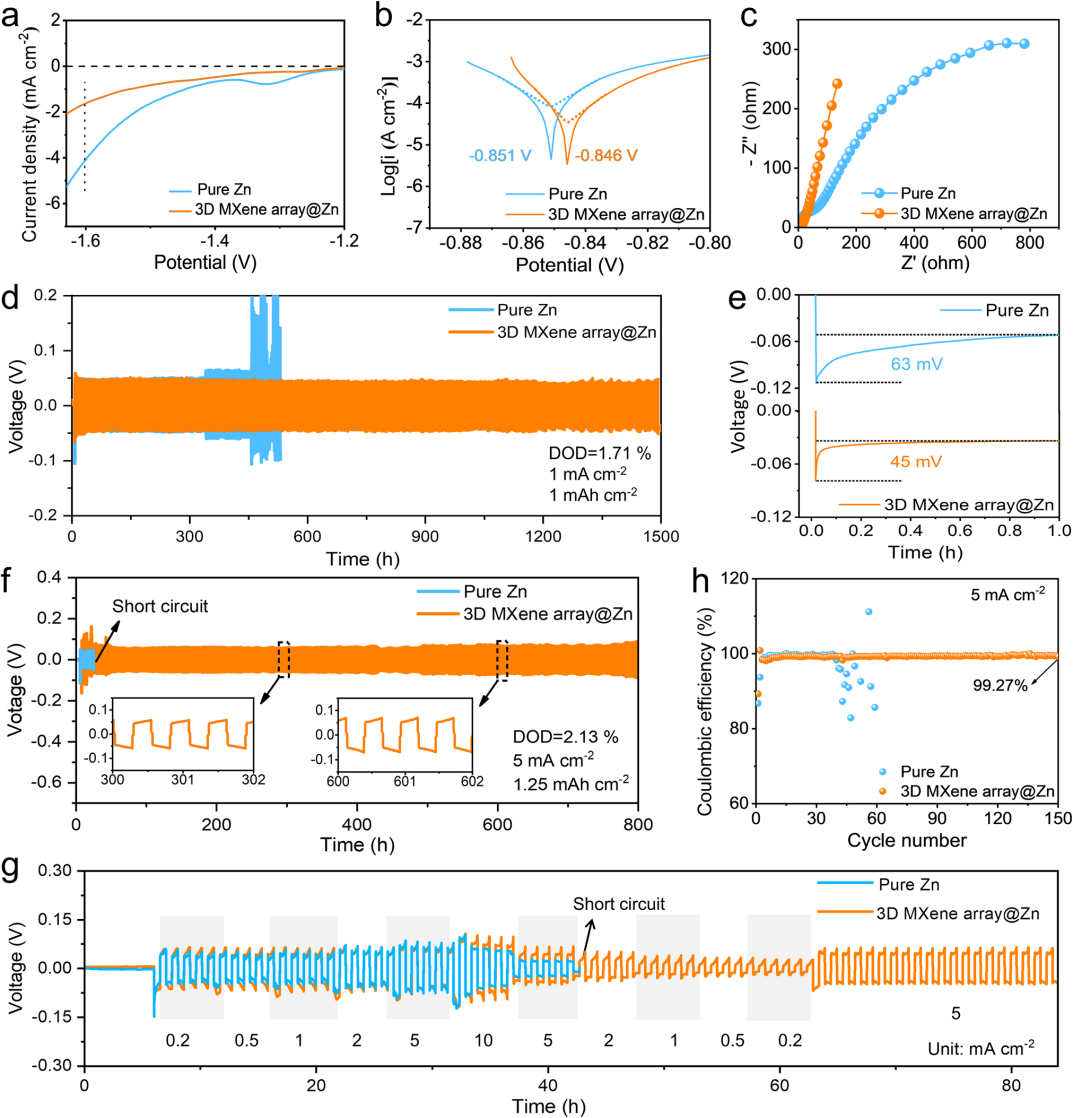
**a** LSV curves of pure Zn and 3D MXene array@Zn in 1 M aqueous Na_2_SO_4_ electrolyte at a scan rate of 1 mV s^−1^. **b** Tafel curves of pure Zn and 3D MXene array@Zn in 3 M Zn(CF_3_SO_3_)_2_ electrolyte at a scan rate of 1 mV s^−1^. **c** EIS spectroscopy of 3D MXene array@Zn (Zn) symmetric cell. **d** Long-term cycling performance of Zn symmetric cell at the condition of 1 mA cm^−2^ and 1 mAh cm^−2^, and **e** corresponding nucleation overpotential. **f** Long-term cycling performance of Zn symmetric cell at the condition of 5 mA cm^−2^ and 1.25 mAh cm^−2^. **g** Rate performance test. **h** Coulombic efficiency of Zn/Cu asymmetric cells with 3D MXene array@Zn anode and pure Zn anode under the current density of 5 mA cm^−2^

Then, the electrochemical performance of pure Zn and 3D MXene array@Zn anode was evaluated by assembling the symmetric cells. Firstly, the cycle performance test of 3D MXene array@Zn electrodes at different thickness case was measured at the current density of 0.5 and 5 mA cm^–2^ (Fig. S6), considering the poor structural stability at 30 µm case and sluggish reaction kinetics at 80 µm case, the optimal thickness of MXene array layer should be 60 µm after balancing the kinetics of interfacial reaction and the stability of interface structure, then all the following tests will be carried out at the optimized condition. Linear sweep voltammetry (LSV) test indicates that the modified electrode has a lower current density of hydrogen evolution in the range from − 1.20 to − 1.63 V (vs Ag/AgCl) in comparison to pure Zn electrode (1.621 mA cm^−2^ for 3D MXene array@Zn and 4.090 mA cm^−2^ for pure Zn at − 1.6 V vs Ag/AgCl) (Fig. 2a). The inhibition of hydrogen evolution reaction (HER) is mainly due to the reduction of direct contact between water molecules in the electrolyte and zinc anode by 3D MXene array interface coating [35–37]. In addition, the Tafel spectra shows that the 3D MXene array@Zn has a higher corrosion potential of − 0.846 V than that of pure Zn anode (− 0.851 V), indicating a lower corrosion reactivity and a lower corrosion rate (Fig. 2b). Meanwhile, it can be calculated that the 3D MXene array@Zn anode exhibits a lower corrosion current density of 0.039 mA cm^−2^ than that of 0.089 mA cm^−2^ for pure Zn anode. Then, the electrochemical impedance spectroscopy (EIS) was performed to study the interfacial transport resistance of the cells (Figs. 2c and S7). It is clear that the 3D MXene array@Zn electrode shows a smaller interface impedance. Such result should be mainly attributed to the excellent conductive and zincophilic properties of the open Ti_3_C_2_T_x_ MXene array interface layer, which allow the efficient transport kinetics at the anode/electrolyte interface [34].

In terms of the cycling performance, the 3D MXene array@Zn-based symmetric cells harvest an outstanding cyclic stability for over 1350 h at the condition of 0.5 mA cm^−2^ and 0.5 mAh cm^−2^ (Fig. S8), over 1500 h at 1 mA cm^−2^ and 1 mAh cm^−2^ (Fig. 2d), as well as over 800 h at 5 mA cm^−2^ and 1.25 mAh cm^−2^ (Fig. 2f). Moreover, the 3D MXene array@Zn electrode also shows a smaller nucleation overpotential of 45 mV than that of the pure Zn anode (63 mV) at the current density of 1 mA cm^−2^ (Fig. 2e). Surprisingly, even at the ultrahigh current density of 20 mA cm^−2^ and a large fixed areal capacity of 10 mAh cm^−2^, such electrode still enables steadily working for 90 h without fluctuation (Fig. S9). However, the Zn symmetric cells show poor cycle stability, and soon get failure as symbolized by short circuit or violent fluctuation of voltage hysteresis. Furthermore, rate performance of the symmetric cell was tested and shown in Fig. 2g. Compared to the pure Zn anode, the engineered Zn anode exhibits stable voltage fluctuation with significantly enhanced reversibility upon the applied current density initially increases from 0.2 to 10 mA cm^−2^, then decline back to 0.2 mA cm^−2^ and finally stay at 5 mA cm^−2^. On the other hand, Zn/Cu asymmetric cells were also assembled. As presented in Fig. 2h, the 3D MXene array@Zn/Cu asymmetric cell exhibits a high reversibility during the Zn plating/stripping cycles, whose coulombic efficiency maintain at 99.27% after 150 cycles. While the Zn/Cu asymmetric cell failed just after 60 cycles. The electrochemical performance of such 3D MXene array@Zn electrode is also demonstrated to being superior to many other previous reports (Fig. S10). However, when such 3D MXene array was served as current collector, the assembled symmetric cell only shows a poor cycle performance (Fig. S11). This is because that the zinc plating/stripping would mainly occur on the surface of deposited zinc rather than the MXene skeleton (Fig. S12), and it is difficult for the MXene skeleton to effectively regulate the deposition behavior.

### Study on Electrochemical Deposition Mechanism of 3D MXene Array@Zn Anode

**Fig. 3:**
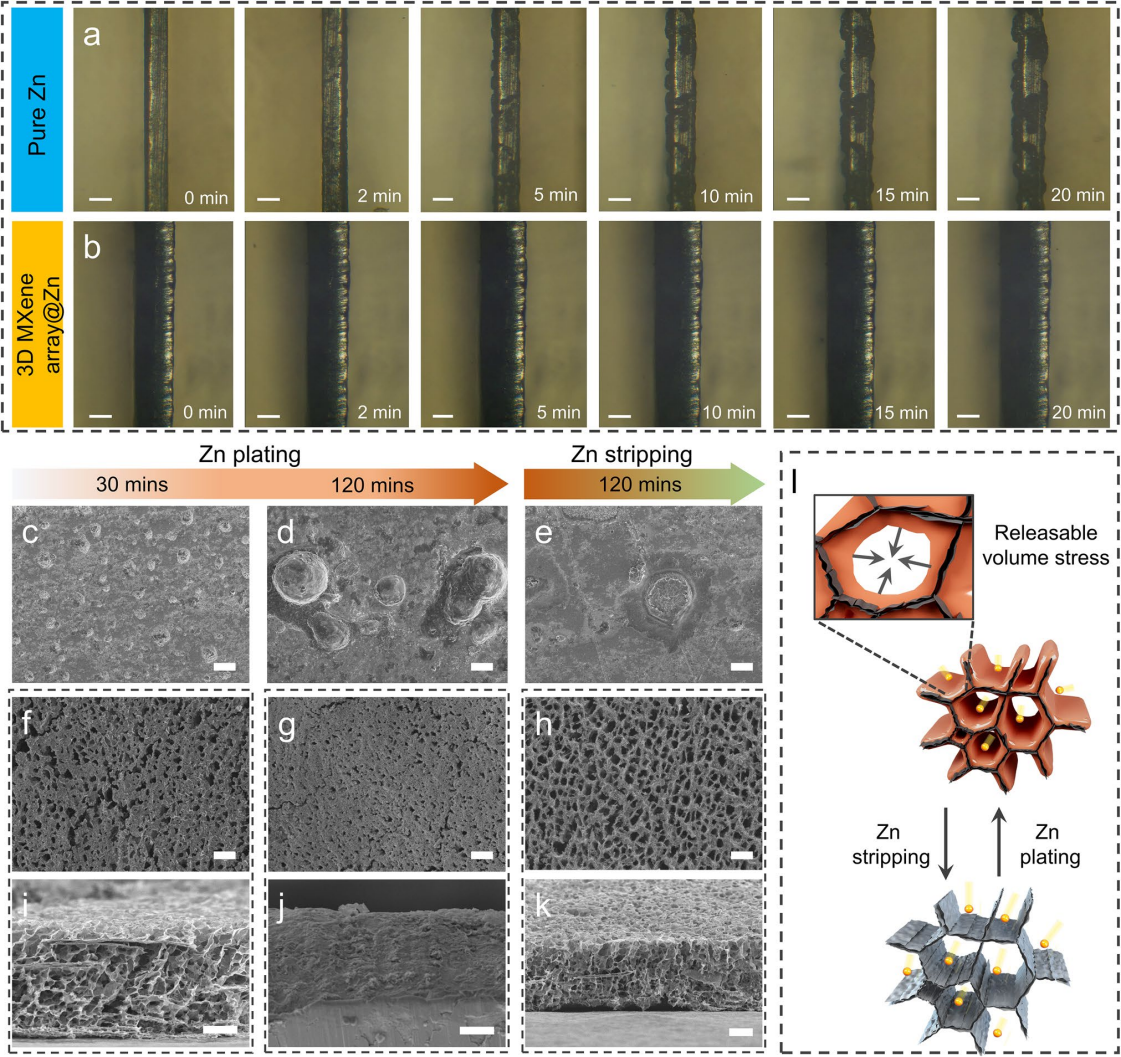
In-situ optical observation of zinc deposition on **a** pure Zn anode and **b** 3D MXene array@Zn anode at different time under the current density of 10 mA cm^−2^ (scale bar = 100 µm). Ex-situ FESEM images of **c–e** pure Zn, **f–h** top-view and **i–k** side-view of 3D MXene array@Zn anode after zinc plating/stripping for different time (scale bar = 10 µm). **l** Schematic diagram of the surface morphology evolution of 3D MXene array@Zn electrode during zinc plating/stripping process

To uncover the origin of the electrochemical performance of Zn metal anode enhanced by the artificial MXene array interface engineering, in-situ optical observation and ex-situ FESEM characterization were applied to record the morphology evolution of zinc planting/stripping behavior on the 3D MXene array@Zn. Optical observation shows that a large amount of small zinc metal particles gradually deposited at the edge and on the surface of pure Zn electrode, then further grow and enveloped into large dendrite with the extension of deposition time to 20 min (Fig. 3a). In sharp contrast to that, the engineered electrode and electrolyte interface enables maintaining clean and without the dendrite formation in the whole process, as depicted in Fig. 3b. Ex-situ FESEM was applied to further reveal the morphological reversibility during zinc planting/stripping process. As shown in Fig. 3c–e, uneven deposition can be observed on the pure Zn electrode surface along with the passing of deposition time, large amount of protuberance sites grown on the surface especially after deposition for 120 min. Moreover, uneven deposition remains even after the full stripping. In a sharp contrast, Fig. 3f–k demonstrate that zinc ion would deposit on the vertically aligned MXene substrate, and the void space of open channels would gradually reduce following with the passing of plating time. Furthermore, those filled channels can be recovered after subsequently stripping for another 120 min. Such result indicates that the open-connected channel of MXene array can release the volume stress during reversible Zn plating/stripping process without structural collapse (Fig. 3l).

**Fig. 4:**
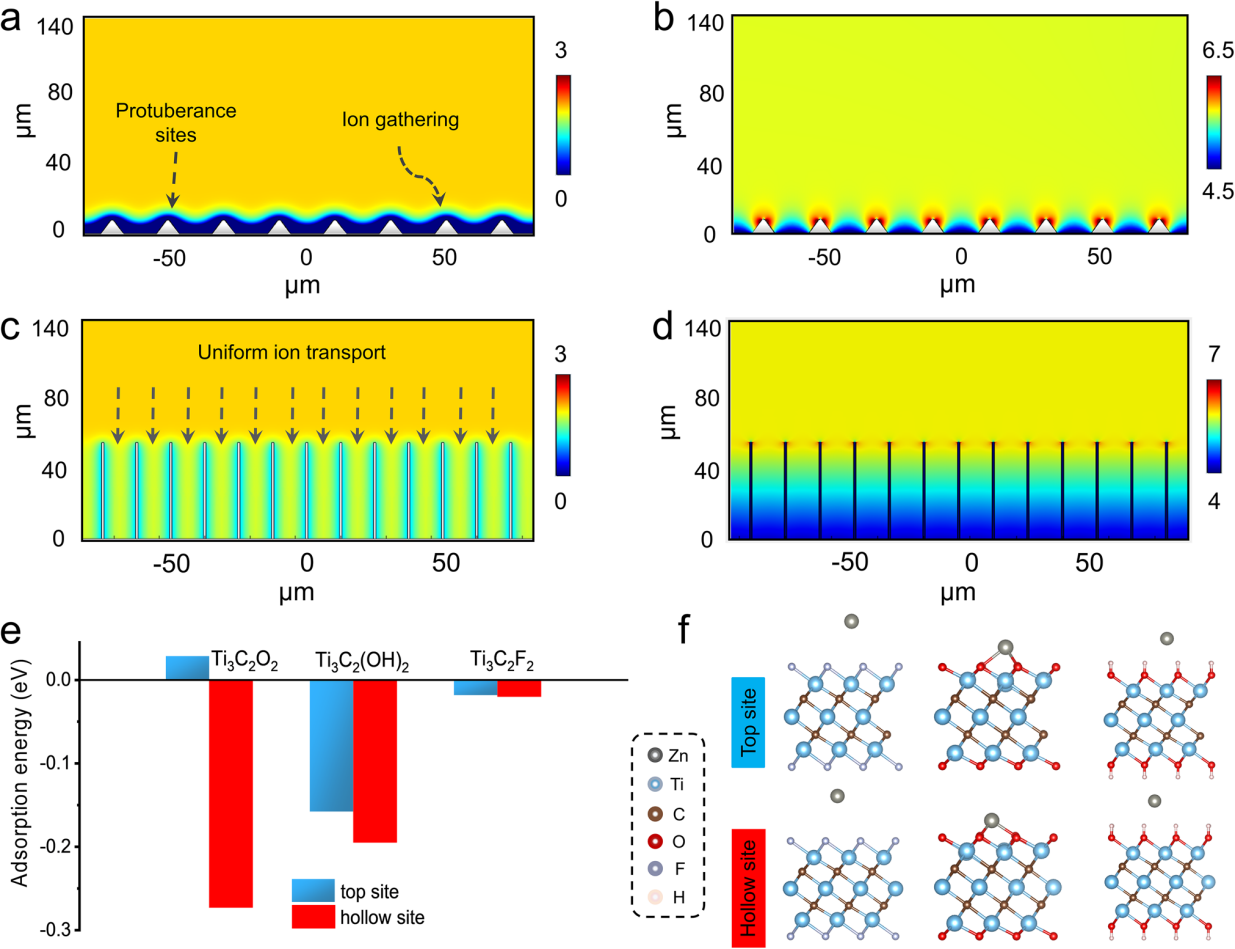
Transient simulation results of zinc ions concentration distribution and electrolyte current density in the **a–b** pure Zn, and **c–d** 3D MXene array@Zn anode at the current density of 0.5 mA cm^−2^. **e** DFT calculation of the absorption energy between single zinc atom and different terminal groups (–F, –O and –OH)-decorated Ti_3_C_2_T_x_ MXene at the hollow and top site, and **f** corresponding side-view diagrams

Then, to explain the ordered deposition of zinc on 3D MXene array@Zn electrode, the dual-field transient simulation was applied to gain insight into the influence of MXene array interface on Zn deposition, including the zinc ion concentration distribution and surface electrolyte current density. The simulation was performed by COMSOL Multiphysics based on a nonlinear phase-field model adopted finite element method in this work, the constructed simulation models are shown in Fig. S13. For pure Zn, the existence of protuberance sites would lead to the ion gathering, making the zinc ion concentration on the anode surface much lower than that in the electrolyte (Fig. 4a). While the vertically aligned array interface can effectively homogenize the zinc ion flux, reduce the zinc ion concentration difference between the zinc anode surface and electrolyte, implying a faster ion transport kinetics (Fig. 4c). In addition, the current density near the protrusion site of pure Zn is much higher than other sites (Fig. 4b), which would easily lead to preferential zinc deposition and finally result in the dendrite formation. For a comparison, the vertical arrays maintain a uniform current density distribution, thus promoting the uniform deposition of zinc ion in the channel (Fig. 4d).

Notably, zinc ion would directly interact with the outermost surficial terminal groups rather than the interior transition metal layers upon depositing on the Ti_3_C_2_T_x_ MXene substrate [38]. Therefore, DFT calculation was carried out to investigate the interaction between Zn atom and Ti_3_C_2_T_x_ MXene decorated with different terminate groups (–O, –OH and –F). Two kinds of adsorption sites (hollow site and top site) were mainly considered in this study, including the hollow site and top site depicted in Fig. S14, where the hollow site is directly above the upper C atom, but the top site is directly above the Ti atom in the outer layer. Detailed calculation results are displayed in Table S1. In general, the result indicates that the hollow sites exhibit a higher adsorption capability for zinc atom than that of top sites. As shown in Fig. 4e–f and Table S1, the adsorption energy of the two adsorption sites of Ti_3_C_2_O_2_ differ greatly, − 0.27 eV at the hollow site and 0.02 eV at the top site. While the adsorption energy of Ti_3_C_2_(OH)_2_ and Ti_3_C_2_F_2_ are − 0.19 eV and − 0.02 eV for the hollow sites, and − 0.15 and − 0.01 eV for the top sites, respectively. Among them, the negative adsorption energy means that the abundant –O and –OH terminate groups on the MXene layer possess a strong capture capability for zinc atom, and it is expected to reduce the zinc nucleation barrier and offer “seed points” for uniform nucleation [39]. However, note that the positive adsorption energy at the top site between Ti_3_C_2_O_2_ and zinc atom should be resulted from the formation of Zn–O bond, which would lead to the distortion of the position of Ti atoms in the subsurface (Fig. S15) and weaken the origin Ti–O bond of Ti_3_C_2_O_2_, thus resulting in a slight increase in the potential energy of the whole system. In sharp contrast to that, the ultra-small adsorption energy in the case of Ti_3_C_2_F_2_ demonstrating that only a weak Van der Waals force between the F terminal group and zinc atom. Based on the calculation results, it can be inferred that the nucleation barrier can be reduced by optimizing the composition of terminal groups. Usually, MXene prepared by different methods has different terminal groups content, and the nucleation barrier can be expressed by the nucleation overpotential. It can be seen from Fig. S16 and Table S2, that the nucleation overpotential of HCl + LiF etched MXene at 0.5 mA cm^−2^ is 13.02 mV, while that of HF etched MXene is 32.55 mV, which verifies the calculation results.

**Fig. 5:**
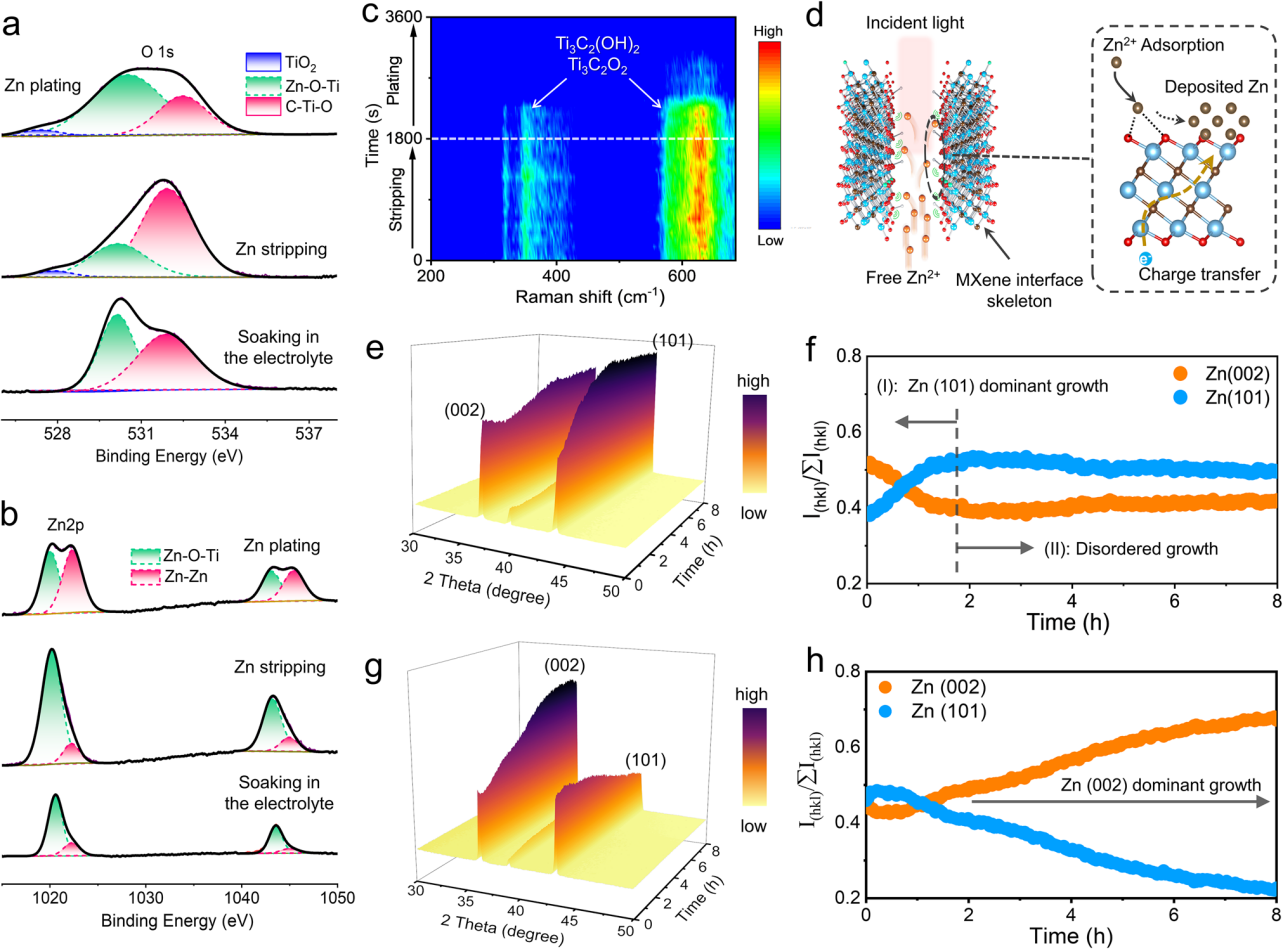
XPS spectra of **a** O 1*s* and **b** Zn 2*p* of 3D MXene array@Zn anode before and after Zn stripping, and then subsequent Zn planting at 0.5 mA cm^−2^. **c** In-situ Raman pattern of 3D MXene array@Zn anode during Zn stripping/planting process at 0.5 mA cm^−2^. **d** Schematic diagram of Zn^2+^ adsorption and deposition on the Ti_3_C_2_T_x_ MXene skeleton during the Raman detection process. In-situ 3D XRD isoline pattern and corresponding proportion changes of (002) and (101) crystal plane for **e–f** pure Zn and **g–h** 3D MXene array@Zn electrode

Then, ex-situ XPS spectrum of O 1*s* and Zn 2*p* of 3D MXene array@Zn anode were studied. For O 1*s* XPS fine spectrum (Fig. 5a), two peaks can be identified after soaking in the electrolyte, including the Zn–O–Ti component at 530.5 eV and C–Ti–O component at 532.5 eV [40, 41]. Note that the existence of Zn–O–Ti component should be resulted from the adsorption of Zn^2+^ onto the terminal groups of Ti_3_C_2_T_x_ MXene during the soaking. Besides, a newly formed small peak at 527.3 eV can be detected during zinc plating/stripping process, which should be attributed to TiO_2_ component owing to the mild oxidation [40]. In contrast to the soaked state, the content of Zn–O–Ti component of 3D MXene array@Zn electrode would decrease after the zinc stripping (22.60%) but significantly increase after the zinc plating (69.21%). While for the Zn 2*p* fine spectrum (Fig. 5b), the Zn–Zn component (1021.5/1044.6 eV) also performs the similar trends as Zn–O–Ti component during the zinc stripping/plating process [18]. Furthermore, in-situ electrochemical Raman spectroscopy was also employed to investigate the deposition mechanism of Zn ion on the Ti_3_C_2_T_x_ MXene substrate. Figure 5c depicts the Raman spectrum of engineered Zn electrode upon stripping and plating process. Among them, the broad peaks located at 349 and 583 cm^−1^ should be attributed to the different vibration modes of Ti_3_C_2_O_2_, the peaks at 383 and 636 cm^−1^ are corresponding to the different vibration modes of Ti_3_C_2_(OH)_2_, while the vibration peaks of Ti_3_C_2_F_2_ locate at 612 and 639 cm^−1^ [42, 43]. In detail, during the zinc stripping process, the intensity of vibration peaks of Ti_3_C_2_O_2_ and Ti_3_C_2_(OH)_2_ would gradually enhance and then maintain stable. However, their intensity would gradually decrease and basically disappear along with the planting time extending. Such phenomenon should be attributed to the nucleation and growth of zinc metal and thus covering on the MXene substrate surface, as illustrated in Fig. 5d. Besides, during the continuous deposition, the wide peak at 350–380 cm^−1^ disappears firstly compared with the wide peak at 610–650 cm^−1^, indicating that the O terminal and OH terminal are the preferential binding sites of zinc ion. Therefore, combined with the results of DFT calculation, ex-situ XPS and in-situ electrochemical Raman spectra, it can be speculated that the zinc atoms would be preferentially adsorbed by the –OH and –O groups of Ti_3_C_2_T_x_ MXene substrate and subsequent deposition on its surface during plating process.

To further reveal the mechanism of MXene array interface on regulating the texture evolution of Zn metal anode, in-situ XRD technology was further applied. In this work, the in-situ XRD measurement of both 3D MXene array@Zn (Fig. S17) and pure Zn electrode (Fig. S18) were carried out under the current density of 0.5 mA cm^−2^ during continue zinc planting for 8 h. For pure Zn, the intensity of Zn(101) crystal plane would initially increase and then tend to stable, while the intensity of Zn(002) would initially decrease then increase and tend to stable (Fig. 5e). Besides, the corresponding proportion change of crystal planes also confirms the dominant growth of Zn(101) plane in the initial stage and following with disordered growth for pure Zn anode (Fig. 5f). Note that the growth of Zn(101) plane is more inclined to induce the dendrite formation owing to its 3D growth direction compared with Zn(002) case (Fig.S19) [16]. In sharp contrast to that, 3D MXene array@Zn electrode performs a highly preferred orientation deposition characteristic, which the intensity of Zn(002) crystal plane gradually increases but the intensity of Zn(101) crystal plane gradually decreases during the zinc deposition (Fig. 5g). As shown in Fig. 5h, the proportion of Zn(002) crystal plane increases from 0.45 to 0.67, while the proportion of Zn(101) crystal plane decreases from 0.45 to 0.22. In addition, comparing the XRD pattern of anodes before and after zinc deposition, the intensity ratio of I_(002)_/I_(101)_ of the 3D MXene array@Zn improves from 0.99 up to 2.95, while the value of pure Zn anode drops from 1.34 to 0.85 (Figs. S20 and S21). Excepting that, the surface morphology of the anodes after continuous deposition for 30 min were also characterized. Figure S22a depicts the FESEM image of 3D MXene array@Zn electrode, which confirms the lateral growth of zinc metal that parallel to the vertically aligned Ti_3_C_2_T_x_ MXene wall and filling the channels, thus achieving the smooth Zn deposition. However, for the pure Zn anode, FESEM image (Fig. S22b) shows that large amount of upright dendrite grown on the surface, implying it the preferred growth along the Zn(101) crystal plane and being consistent with the in-situ XRD result.

**Fig. 6:**
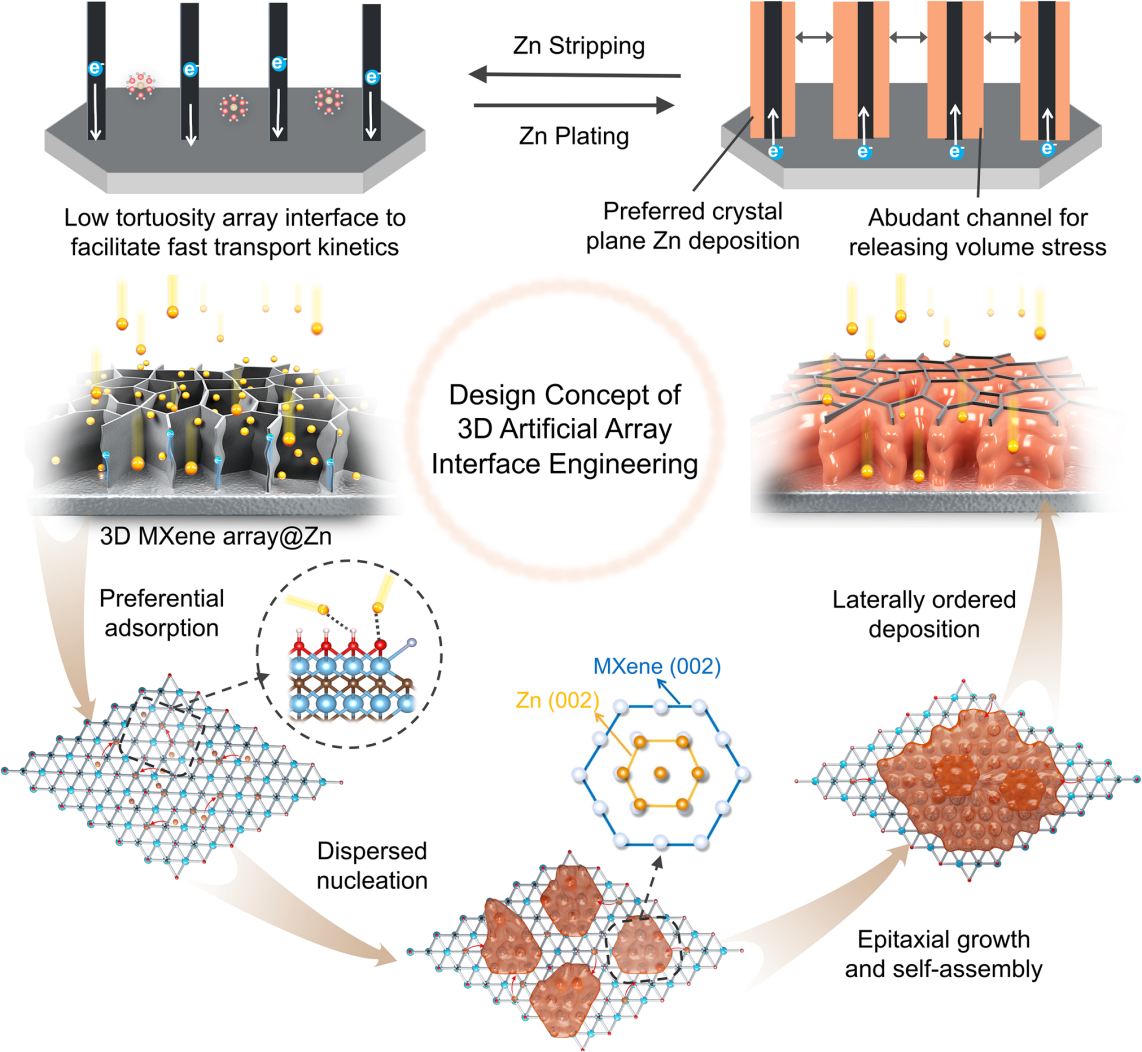
Schematic diagram of 3D artificial array interface engineering to enabling volume stress releasable, preferred orientation growth and dendrite-free stable Zn metal anode

According to the results of above theoretical and experimental investigation, the mechanism of 3D artificial array interface on modulating the growth kinetics and deposition behavior of Zn atoms can be basically described in Fig. 6. In terms of deposition behavior, in the initial plating state, Zn ion rapidly diffuse along the low tortuosity channel and being adsorbed onto the MXene surface, while the electrons are simultaneously transferred through the conductive skeleton, promoting the nucleation of Zn ion on it. Then, during the further plating process, deposited zinc would laterally grow along the MXene substrate. Note that those array channels can release the volume stress. While for the growth kinetics, in the initial stage, vertically aligned MXene array is expected to facilitate the ion transport yet homogenize the Zn ion flux and current density distribution, and Zn ion would be preferentially adsorbed by the oxygen-contained terminate groups (–O, –OH) on the surface of Ti_3_C_2_T_x_ MXene skeleton. Subsequently, dispersed nucleation of Zn ion would occur on the skeleton owing to its high zincophilic and conductive properties of MXene substrate. Moreover, benefited from the high Zn(002) lattice compatibility of highly exposed MXene (002) substrate, those as-dispersed nucleation would tend to epitaxial growth, self-assembly and gradually cover the MXene substrate. Finally, subsequent Zn atoms would deposit along the Zn(002) plane layer-by-layer and realizing the dendrite-free deposition.

### Application of 3D MXene Array@Zn Anode in Aqueous Zn-Ion Batteries

**Fig. 7:**
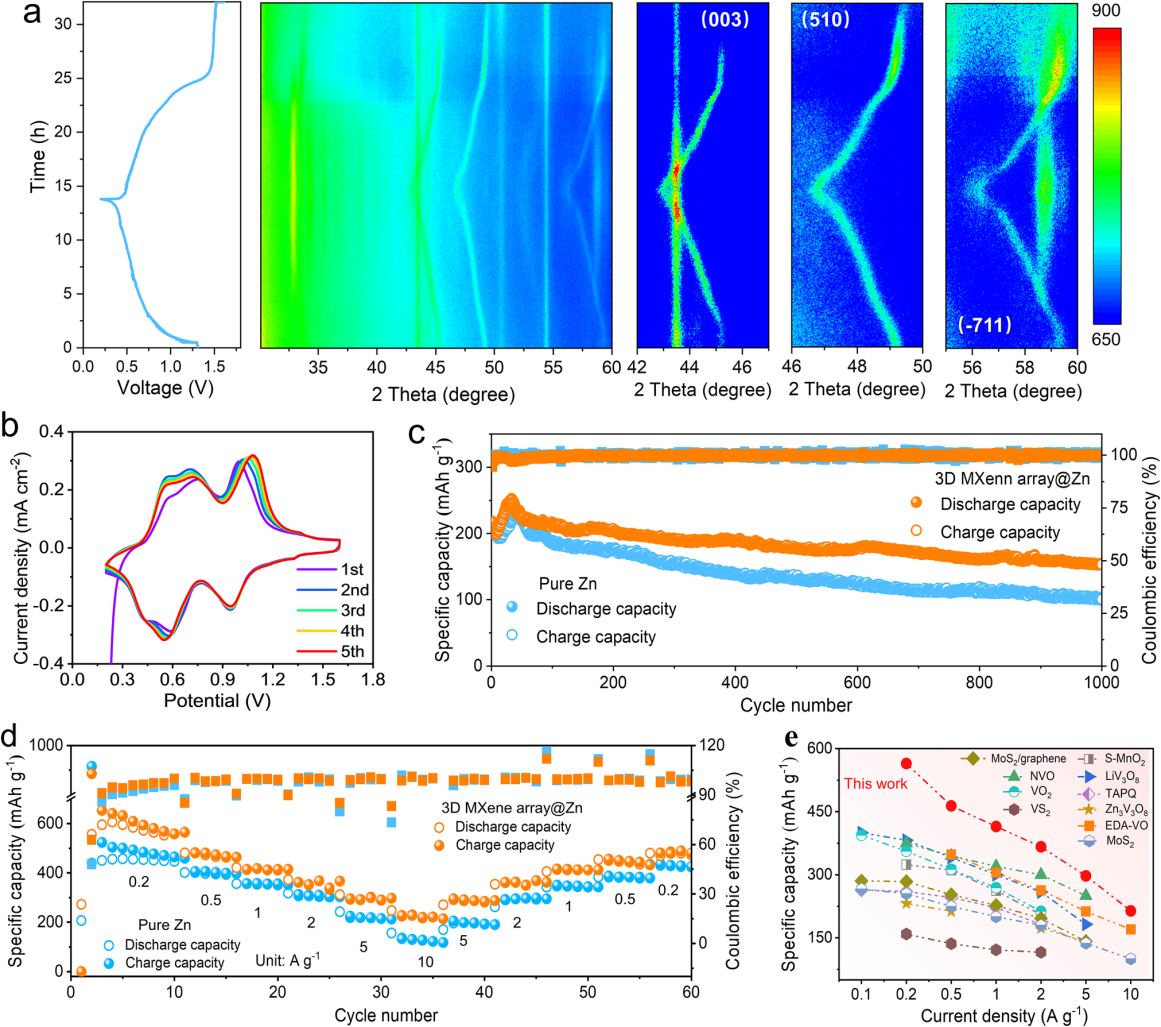
**a** In-situ XRD pattern of Zn/VO_2_ battery at the condition of charge–discharge cycle at the current of 0.5 mA. **b** CV curves of the initial five cycles of the 3D MXene array@Zn/VO_2_ battery at the scan rate of 0.2 mV s^−1^. **c** Long-term cycling performance at the current density of 5 A g^−1^. **d** Rate performance of Zn/VO_2_ battery at various current density. **e** Comparison of the electrochemical performance with other previous works

In order to evaluate the practical application of 3D MXene array@Zn anode for AZIBs, both of Zn/VO_2_ and 3D MXene array@Zn/VO_2_ batteries were respectively assembled. VO_2_ cathode was synthesized via a facile hydrothermal method (Fig. S23). At first, in-situ XRD technique was carried out to investigate the storage mechanism. As shown in Fig. 7a, the diffraction peak of (003), (510) and (− 711) of VO_2_ would shift toward a lower angel in the charge process, and recovery during the discharge process, which indicates the reversible insertion/extraction of zinc ion from VO_2_ [44]. Note that the diffraction peak located at about 33° should be attributed to the formation of Zn_3_(OH)_2_V_2_O_7_·2H_2_O by-product (PDF: 00-050-0570), which was generated in the discharge process and dissolved in the subsequent charge process [45]. On the other hand, the EIS curves (Fig. S24) also indicate that the MXene array interface coating enables reducing the transport resistance. Then, cyclic voltammogram (CV) curves of the 3D MXene array@Zn/VO_2_ and Zn/VO_2_ batteries at the initial five cycles were depicted in Figs. 7b and S25, respectively. It is clear that they possess similar redox peaks located at approximately 0.43/0.56 V, 0.59/0.75 V and 0.94/1.00 V. After that, the cyclic stability of the cells was evaluated. Note that the 3D MXene array@Zn/VO_2_ battery can still deliver a capacity of 380 mAh g^−1^ after 100 cycles at the 0.5 A g^−1^, much higher than that of 136 mAh g^−1^ for the Zn/VO_2_ one (Fig. S26). Furthermore, such 3D MXene array@Zn-based battery also displays a higher discharge capacity and capacity retention at the high current density of 5 A g^−1^. As shown in Fig. 7c, it can retain a discharge capacity of 153.6 mAh g^−1^ after 1000 cycles with the capacity retention of 77.2%, compared with the capacity of 97.5 mAh g^−1^ with the capacity retention of 56.2% for pure Zn/VO_2_ system. It should be pointed that the ascending stage of specific capacity in the initial cycles was mainly attributed to the electrochemical activation of active material. Excepting that, the result of rate performance (Fig. 7d) demonstrates that the 3D MXene array@Zn/VO_2_ battery possesses a better rate capability with the discharge capacities of 564.6, 463.8, 414.7, 366.4, 297.2, 214.1 mAh g^−1^ obtained at 0.2, 0.5, 1, 2, 5 and 10 A g^−1^, respectively, which is much higher than that of the counterpart one. Notably, such achieved electrochemical performance is also superior to many other previous reports, as shown in Fig. 7e [46–55]. Therefore, it has been demonstrated that the rate capability and cyclic stability of Zn/VO_2_ battery can be significantly improved by adopting such engineered anode.

## Conclusion

In summary, the concept of 3D artificial array interface engineering has been demonstrated to be an effective solution to control the Zn deposition, achieving the eliminated volume stress, preferred orientation growth and dendrite-free Zn metal anode. According to the comprehensive studies of theoretical calculation and experimental characterizations, 3D open MXene array interface can be expected to facilitate the ion transport, homogenize the Zn ion flux, and induce the preferred orientation deposition along the Zn (002) crystal plane. Besides, the resistance of electrochemical corrosion and HER of the electrode also can be enhanced. When such engineered electrode was applied to aqueous Zn-ion batteries, the assembled symmetric cell exhibits excellent rate capability and cyclic stability, which can steadily operate for over 1350 h at 0.5 mA cm^−2^ and 0.5 mAh cm^−2^, 1500 h at 1 mA cm^−2^ and 1 mAh cm^−2^, 800 h at 5 mA cm^−2^ and 1.25 mAh cm^−2^, and 90 h even at 20 mA cm^−2^ and 10 mAh cm^−2^, respectively. In addition, compared with the pure Zn anode system, the as-constructed 3D MXene array@Zn/VO_2_ batteries also show an enhanced cyclic stability (153.6 mAh g^−1^ after 1000 cycles at 5 A g^−1^) and rate capability. Therefore, such 3D open array interface engineering strategy is highly anticipated to offer a new insight into the development of stable Zn metal anode and high-performance aqueous Zn-ion batteries.

### Supplementary Information

Below is the link to the electronic supplementary material.

Supplementary file1 (PDF 1797 KB)
