# Supplementary material for: 3D Artificial Array Interface Engineering Enabling Dendrite-Free Stable Zn Metal Anode
Source: Nanomicro Lett. 2023 Jan 17;15:37. doi: 10.1007/s40820-022-01007-z (PMC9845508; doi:10.1007/s40820-022-01007-z)
Supplement: Supplementary file 1 — Supplementary file1 (PDF 1797 KB) [file 40820_2022_1007_MOESM1_ESM.pdf]

Supporting Information for

3D Artificial Array Interface Engineering Enabling Dendrite-Free Stable Zn Metal Anode

Jianbin Ruan[1], Dingtao Ma[1, 2, *], Kefeng Ouyang[1], Sicheng Shen[1], Ming Yang[1], Yanyi Wang[1], Jinlai Zhao[3], Hongwei Mi[1, 4], Peixin Zhang[1, 4, *]

[1] College of Chemistry and Environmental Engineering, Shenzhen University, Shenzhen 518060, P. R. China

[2] Institute of Microscale Optoelectronics, Shenzhen University, Shenzhen 518060, P. R. China

[3] College of of Materials Science and Engineering, Shenzhen University, Shenzhen 518060, P. R. China

[4] Guangdong Flexible Wearable Energy and Tools Engineering Technology Research Center, Shenzhen 518060, P. R. China

*Corresponding authors. E-mails: mdt2500@szu.edu.cn (Dingtao Ma); pxzhang@szu.edu.cn (Peixin Zhang)

Supplementary Figures

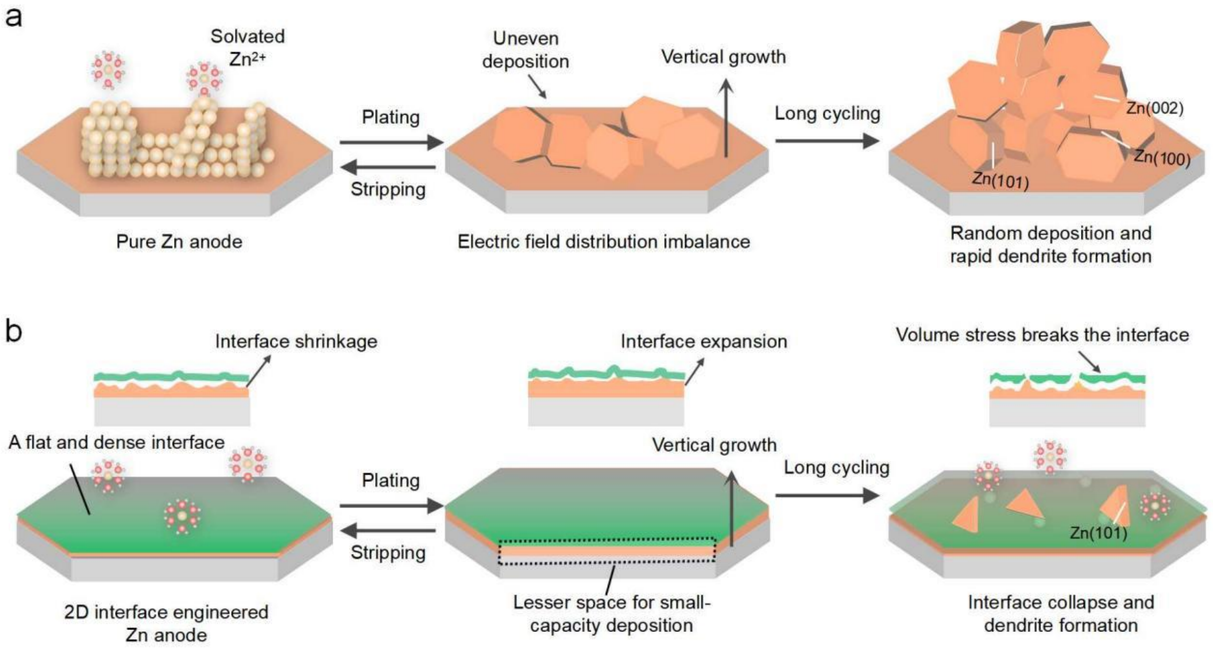

Fig. S1 Schematic illustration of structural evolution of (**a**) pure Zn anode and (**b**) 2D interface engineered Zn anode after long-term of plating and stripping cycling

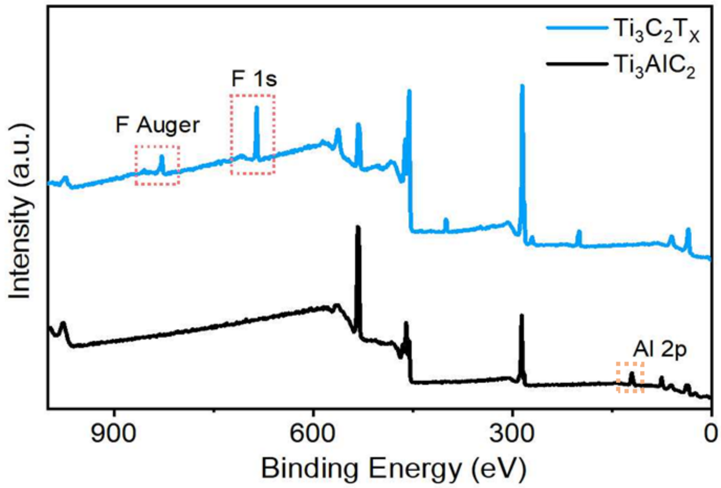

Fig. S2 XPS spectra of the as-synthesized $Ti_3C_2T_x$ MXene and Ti_3AlC_2 powder

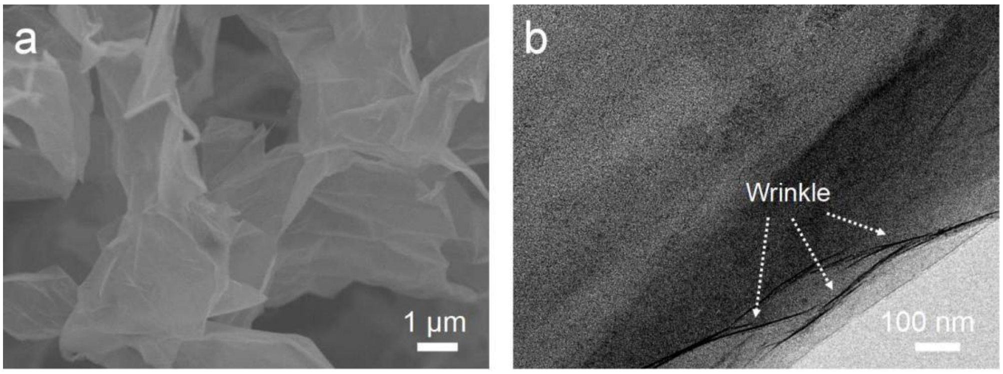

Fig. S3 (**a**) FESEM image and (**b**) corresponding TEM image of the $Ti_3C_2T_x$ MXene nanosheets

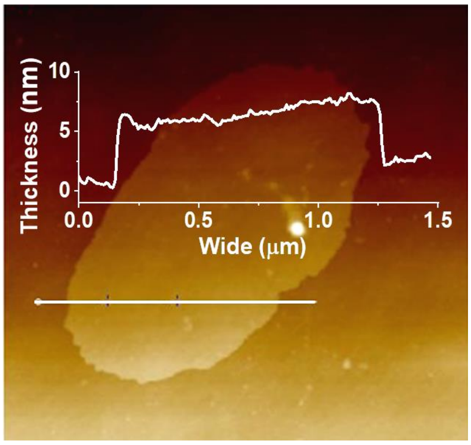

Fig. S4 AFM image of the as-synthesized $Ti_3C_2T_x$ MXene nanosheets

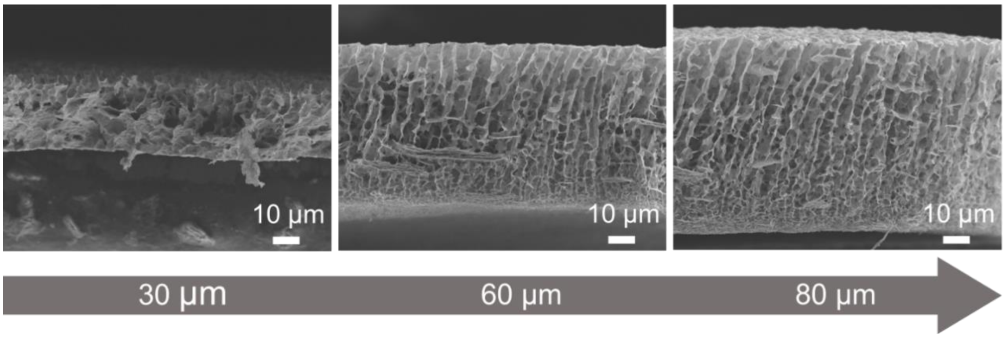

Fig. S5 Side-view FESEM images of the 3D MXene array interface with different thickness of (**a**) 30 μm, (**b**) 60 μm, and (**c**) 80 μm

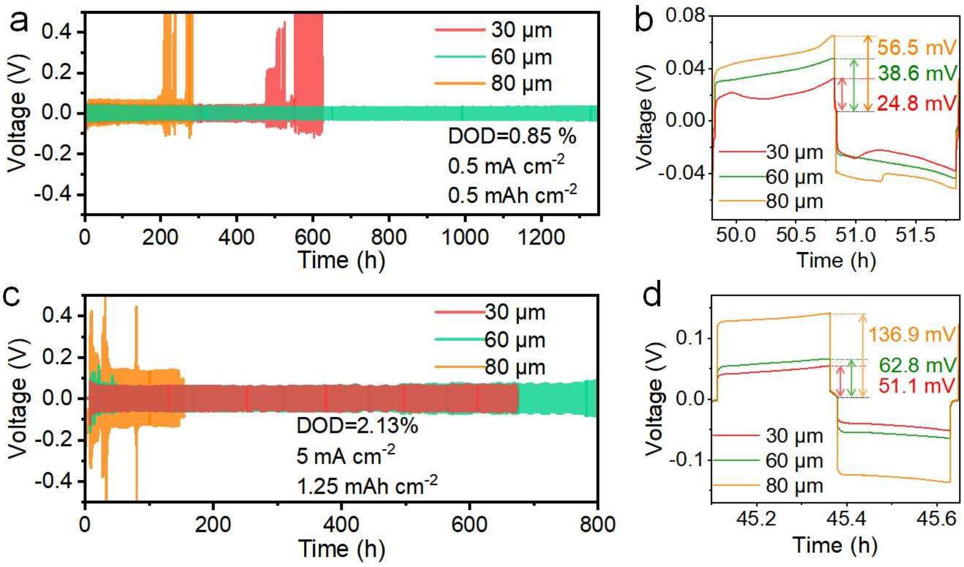

Fig. S6 The galvanostatic cycling of symmetrical 3D MXene array@Zn with different thickness (30, 60 and 80 μm) and corresponding partially enlarged view at (**a, b**) 0.5 mA cm^{-2} with a fixed areal capacity of 0.5 mAh cm^{-2}, and (**c, d**) 5 mA cm^{-2} with a fixed areal capacity of 1.25 mAh cm^{-2}

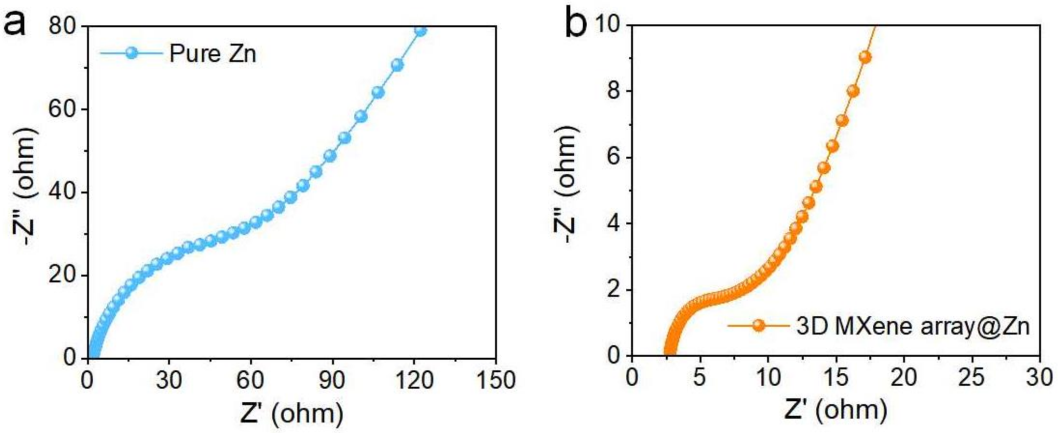

Fig. S7 Partial enlargement in the high-medium frequency of EIS curves in Figure 2c. (**a**) Pure Zn, (**b**) 3D MXene array@Zn

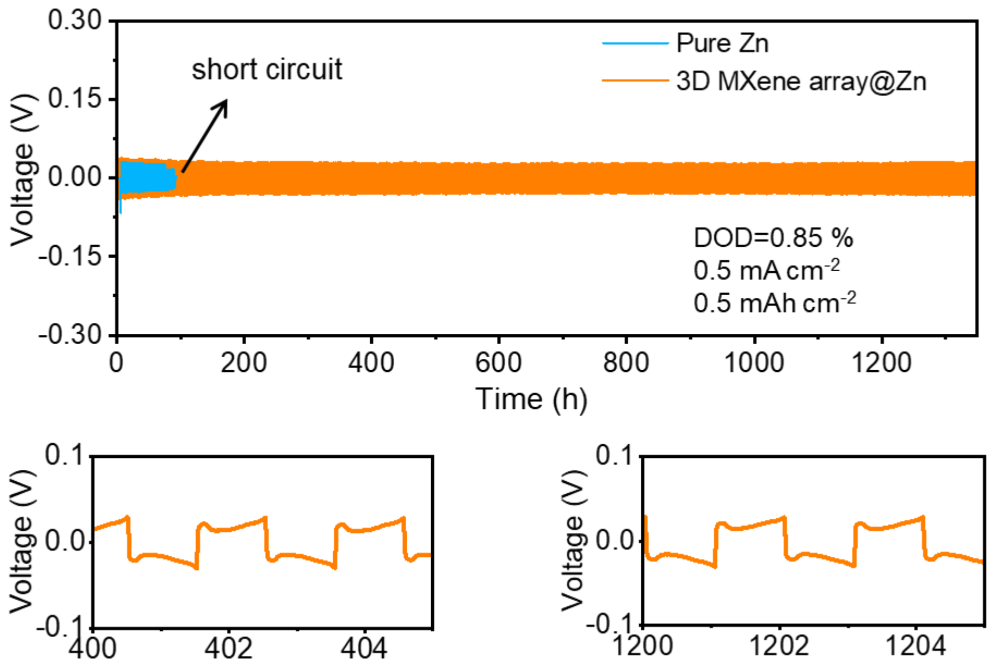

Fig. S8 The galvanostatic cycling of symmetrical 3D MXene array@Zn and Zn cells at 0.5 mA cm^{-2} with a fixed areal capacity of 0.5 mAh cm^{-2}

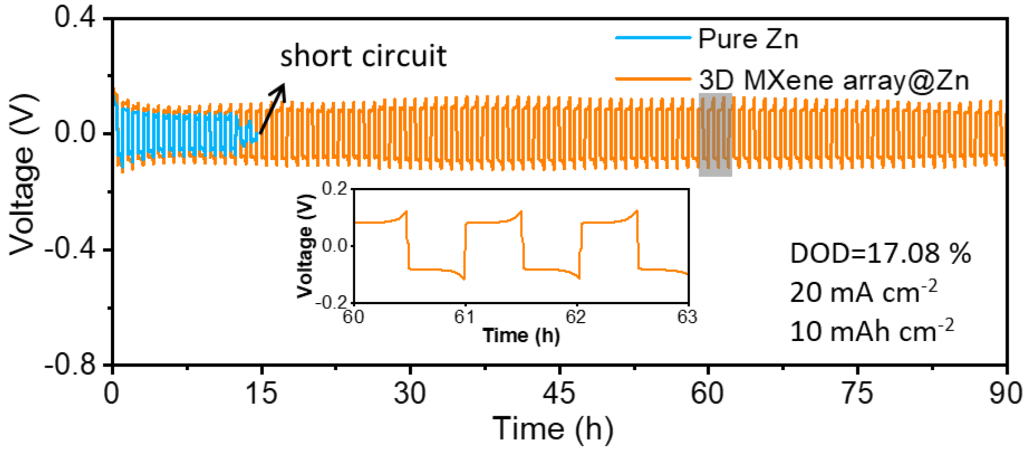

Fig. S9 The galvanostatic cycling of symmetrical 3D MXene array@Zn and Zn cells at 20 mA cm^{-2} with a fixed areal capacity of 10 mAh cm^{-2}

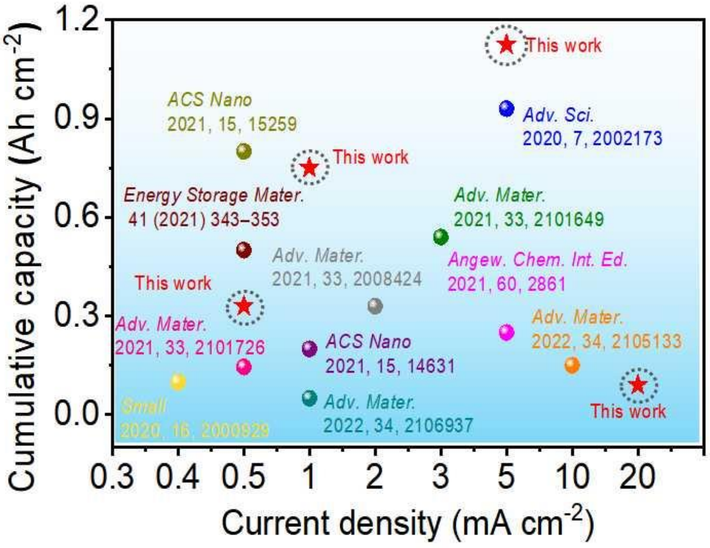

Fig. S10 The comparison of electrochemical performance between this work and other previous reports [S1-S9]

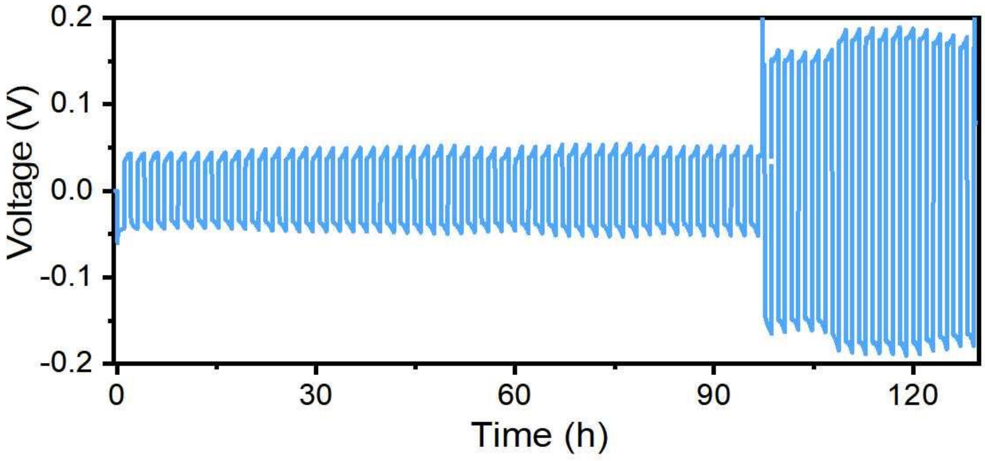

Fig. S11 Cycle performance of symmetric cell assembled by the electrode that zinc deposited on the 3D MXene array current collector at the condition of 1 mA cm^{-2} and 1 mAh cm^{-2}

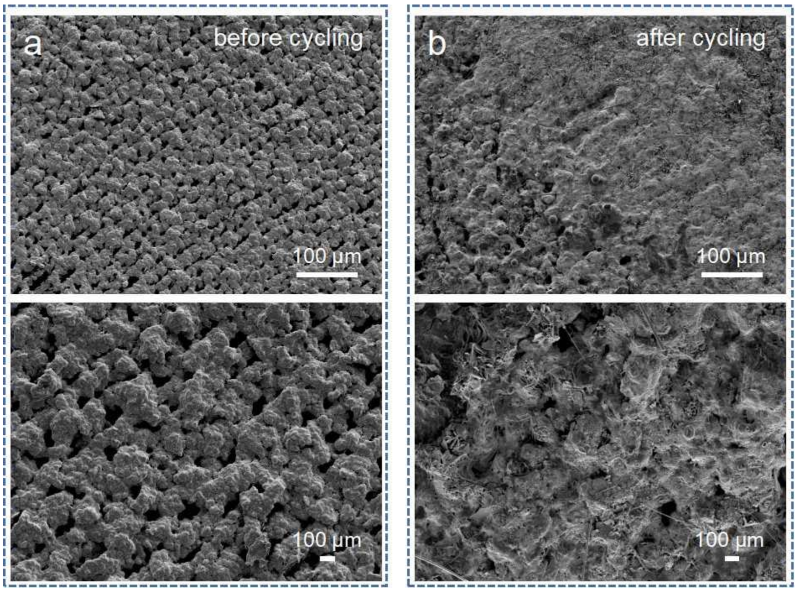

Fig. S12 FESEM images of electrode that zinc deposited on the 3D MXene array current collector. (**a**) before, and (**b**) after cycling for 100 h

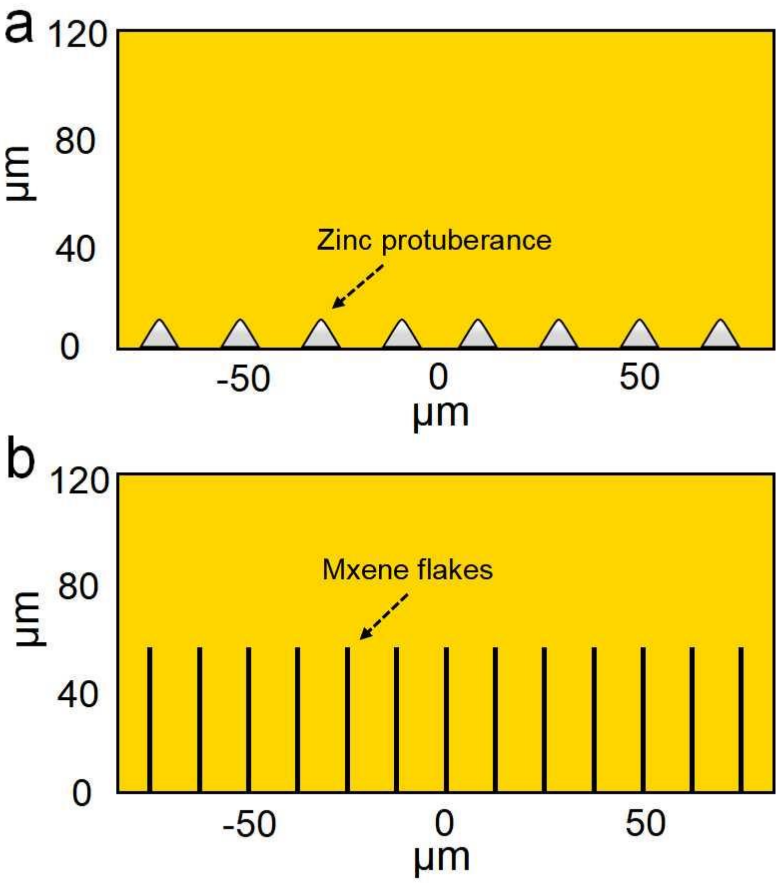

Fig. S13 Simulation models for the (**a**) pure Zn anode, and (**b**) 3D MXene array@Zn anode

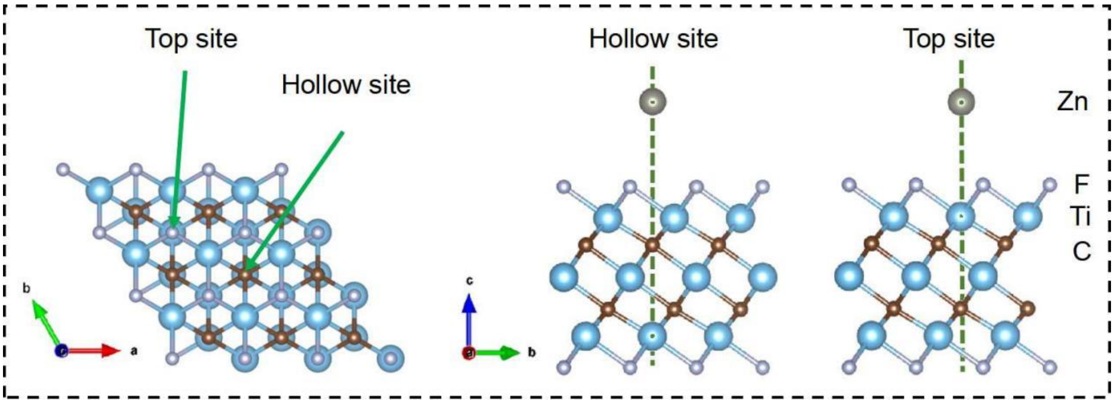

Fig. S14 Schematic diagram of the hollow site and top site on the $Ti_3C_2T_x$ MXene

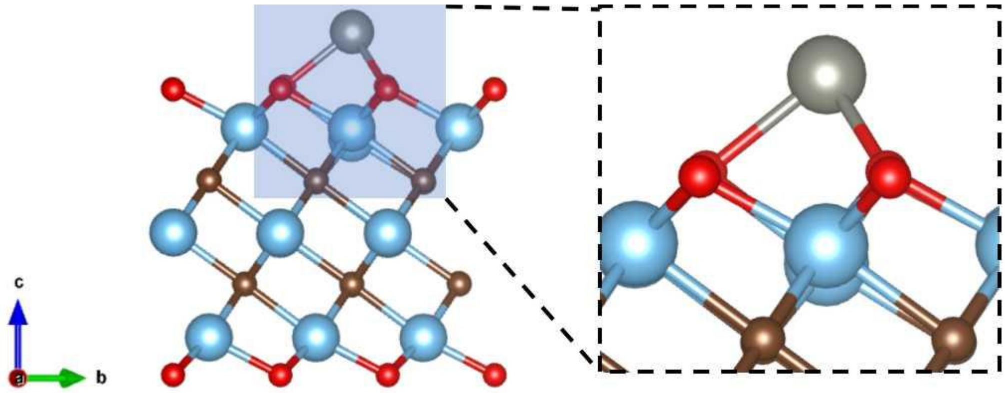

Fig. S15 Distortion of the position of Ti atoms in the subsurface when Zn atom adsorption on the top site of $Ti_3C_2O_2$ MXene

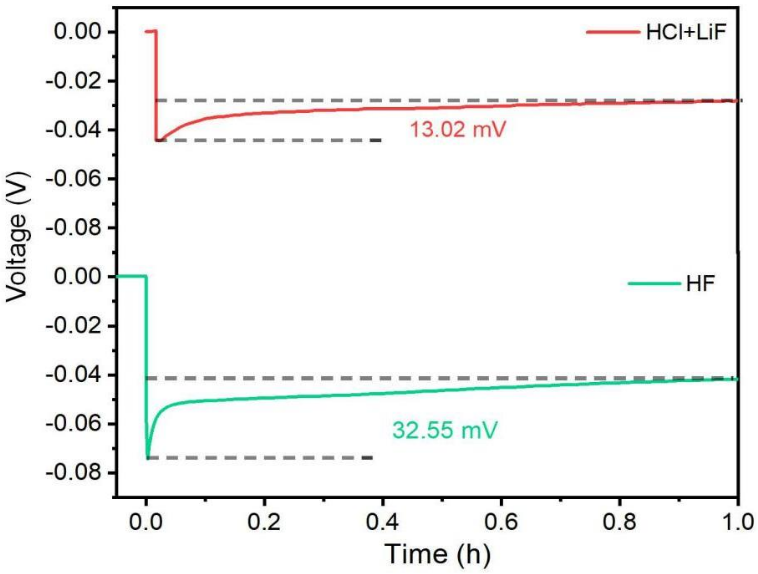

Fig. S16 The nucleation overpotential of 3D MXene array interface engineered Zn metal anodes with different terminal groups content at the current density of 0.5 mA cm^{-2}

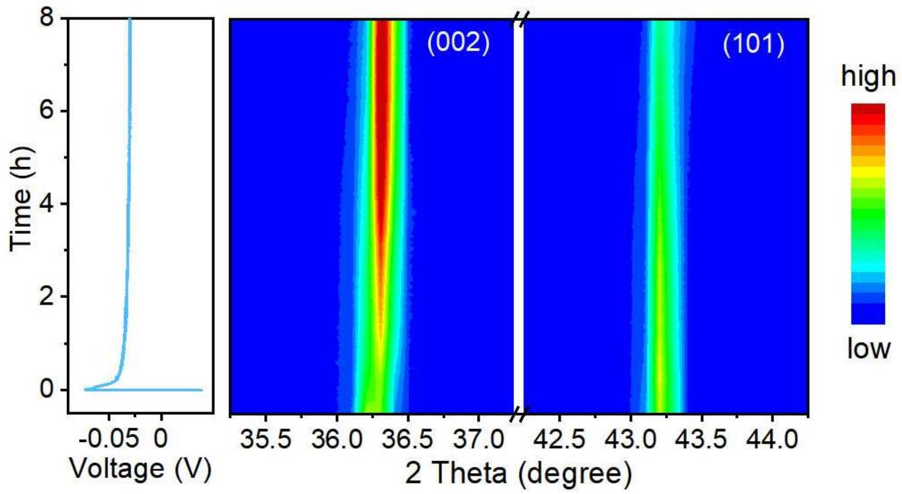

Fig. S17 In-situ 2D XRD pattern of 3D MXene array@Zn at the condition of continuous electroplating of zinc ions at a current density of 0.5 mA cm^{-2}

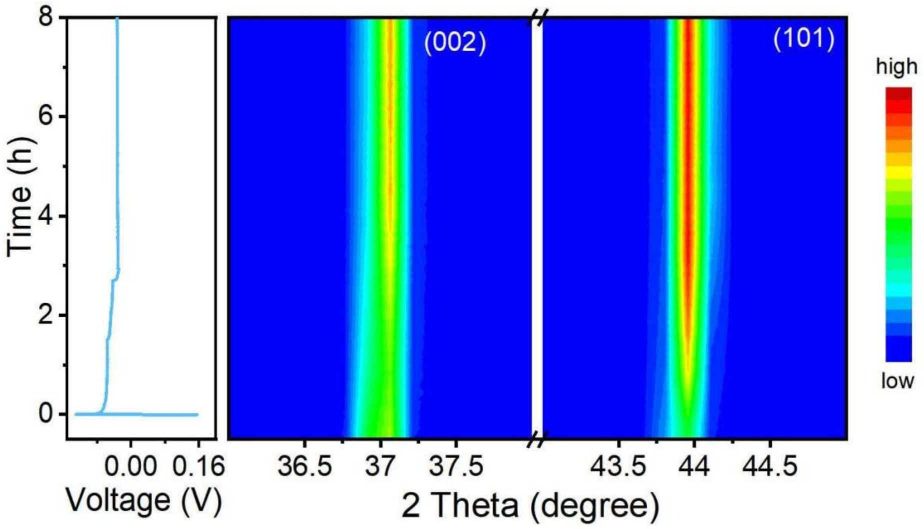

Fig. S18 In-situ 2D XRD pattern of pure Zn anode at the condition of continuous electroplating of zinc ions at a current density of 0.5 mA cm^{-2}

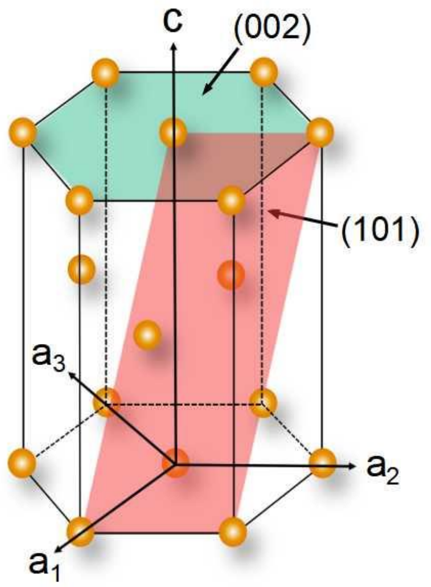

Fig. S19 Crystal structure of Zn metal

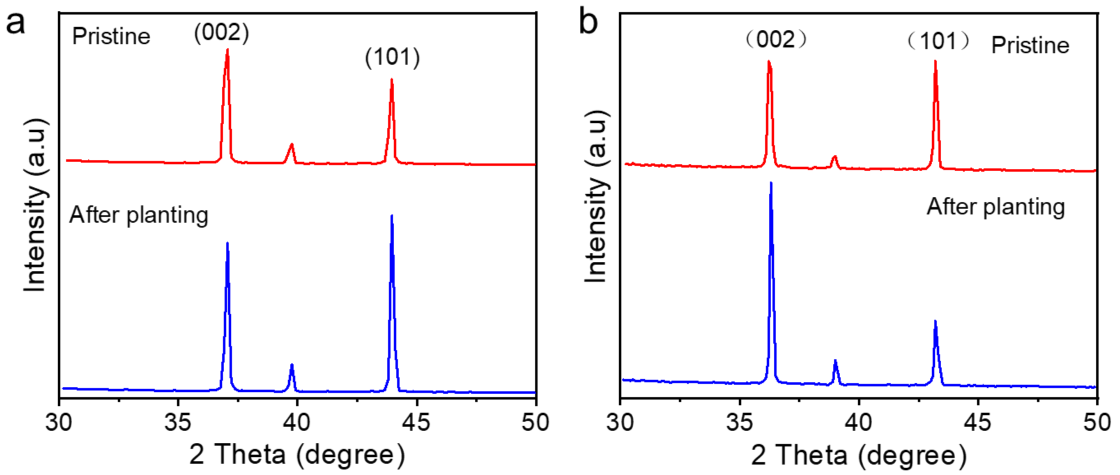

Fig. S20 XRD pattern of different stages in the in-situ measurement (continues Zn planting for 8 h at a current density of 0.5 mA cm^{-2}) of (**a**) pure Zn anode, and (**b**) 3D MXene array@Zn anode

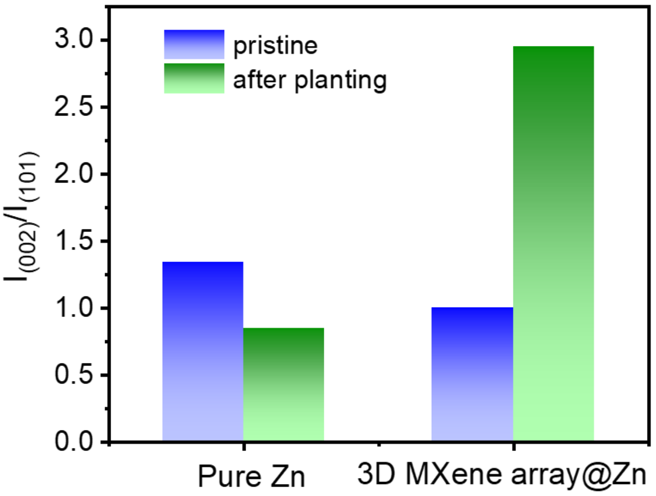

Fig. S21 $I_{(002)}/I_{(101)}$ ratio of different stages in the in situ measurement (continues Zn planting for 8 h at a current density of 0.5 mA cm^{-2}) of 3D MXene array@Zn and pure Zn anode

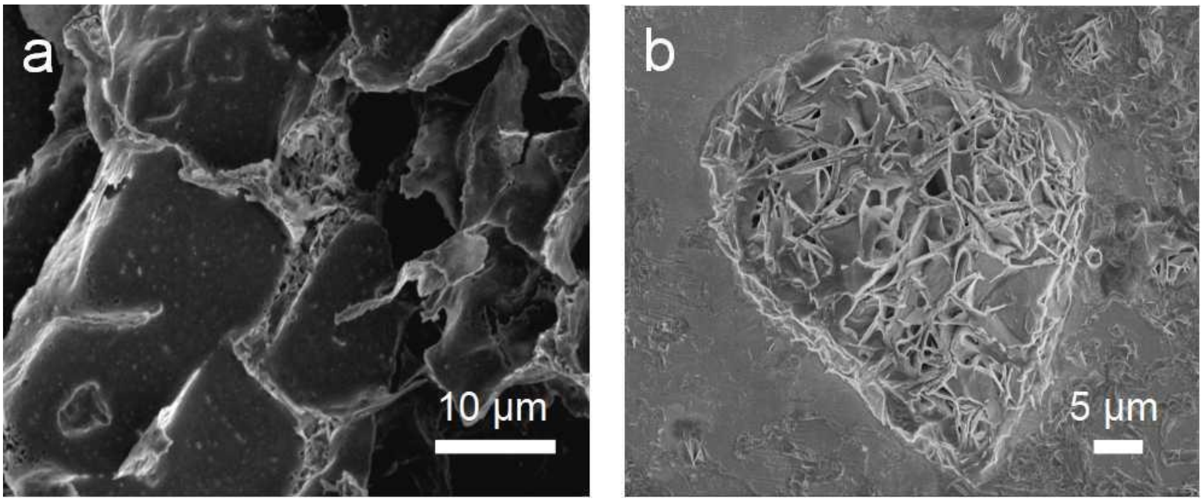

Fig. S22 FESEM images of (**a**) 3D MXene array@Zn and (**b**) pure Zn anodes after continuous deposition for 30 mins

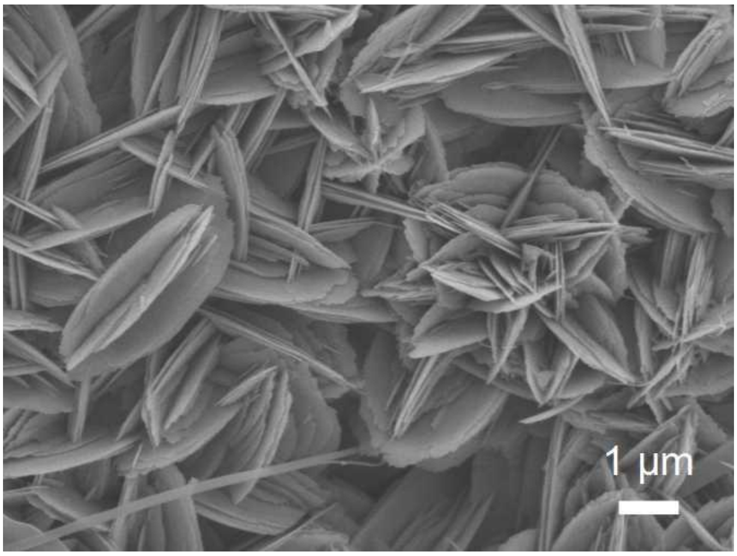

Fig. S23 FESEM image of the VO_2 cathode material

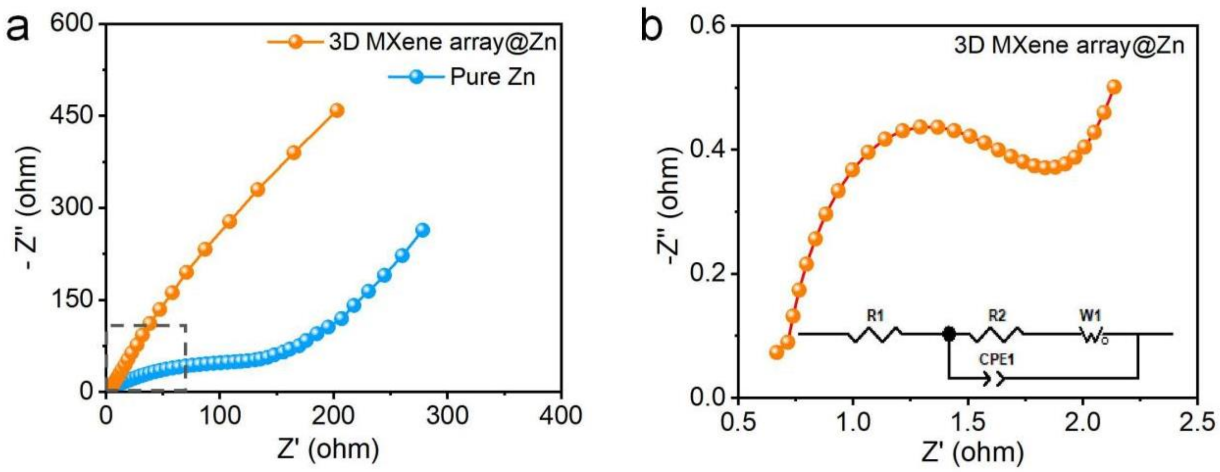

Fig. S24 (**a**) EIS curves of 3D MXene array@Zn/VO_2 and Zn/VO_2 batteries, (**b**) the corresponding enlarged EIS curve of 3D MXene array@Zn/VO_2 battery

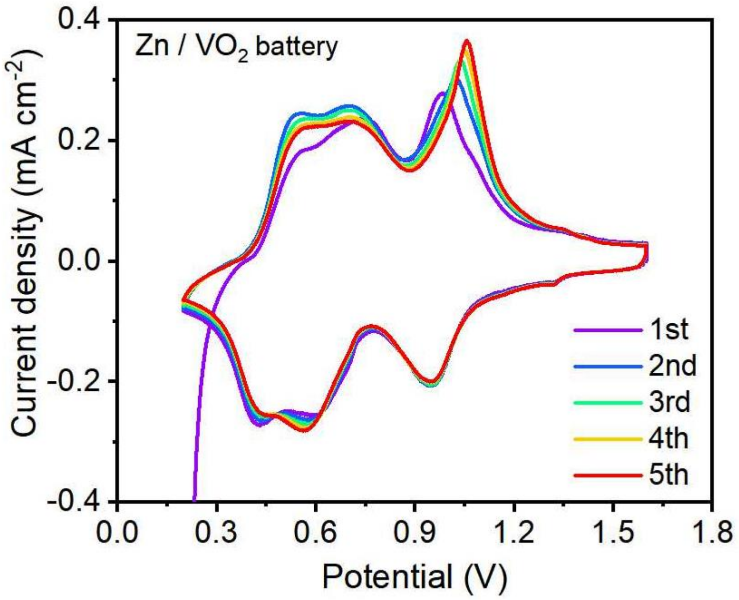

Fig. S25 CV curves of the initial five cycles of Zn/VO_2 battery at the scan rate of 0.2 mV s^{-1}

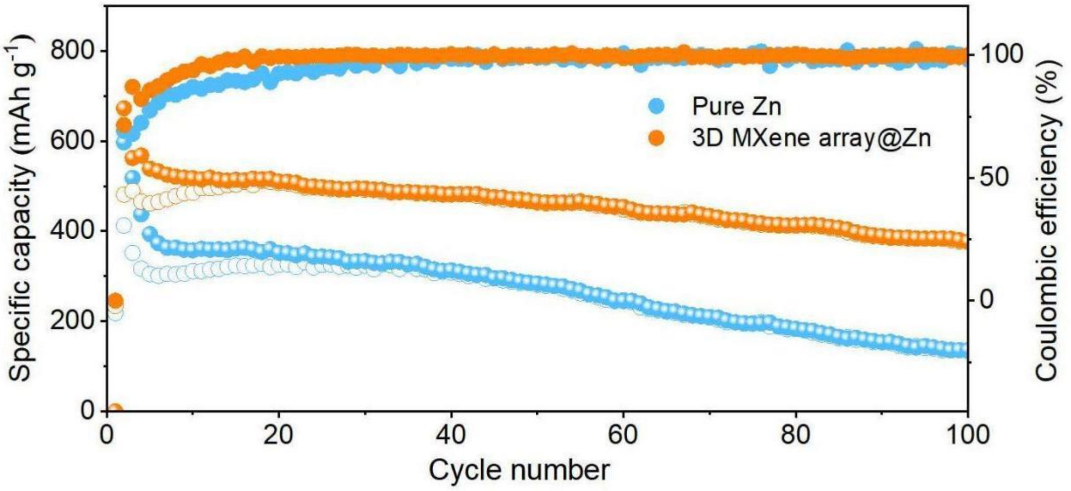

Fig. S26 Long-term cycling performance of 3D MXene array@Zn/VO_2 and pure Zn/VO_2 batteries at the current density of 0.5A g^{-1}

Table S1 DFT calculation results of the adsorption energy between zinc atom and $Ti_3C_2T_x$ MXene (x=-O, -OH and -F)

		E_{tot}	E_{base}	E_{mol}	E_{ads}
MXene-O	top site	-573.122	-573.143	-0.00786	0.028444
	hollow site	-573.423	-573.143	-0.00786	-0.27272
MXene-OH	top site	-631.665	-631.499	-0.00786	-0.15809
	hollow site	-631.701	-631.499	-0.00786	-0.19462
MXene-F	top site	-525.224	-525.198	-0.00786	-0.01826
	hollow site	-525.225	-525.198	-0.00786	-0.01981

Table S2 The content of different terminal groups of MXene synthesized by different methods (O contain both of -O and -OH)

	HF etching (weight %)	HCl+LiF etching (weight %)
O	30.57	49.06
F	68.48	48.45
Cl	0.95	2.49

Supplementary References

[S1] X. Liu, F. Yang, W. Xu, Y. Zeng, J. He et al., Zeolitic imidazolate frameworks as Zn^{2+} modulation layers to enable dendrite-free Zn anodes. Adv. Sci. **7**(21), 2002173 (2020). https://doi.org/10.1002/advs.202002173

[S2] J. Zhou, M. Xie, F. Wu, Y. Mei, Y. Hao et al., Ultrathin surface coating of nitrogen-doped graphene enables stable zinc anodes for aqueous zinc-ion batteries. Adv. Mater. **33**(33), 2101649 (2021). https://doi.org/10.1002/adma.202101649

[S3] S. Li, J. Fu, G. Miao, S. Wang, W. Zhao et al., Toward planar and dendrite-free Zn electrodepositions by regulating Sn-crystal textured surface. Adv. Mater. **33**(21), 2008424 (2021). https://doi.org/10.1002/adma.202008424

[S4] X. Li, Q. Li, Y. Hou, Q. Yang, Z. Chen et al., Toward a practical Zn powder anode: $Ti_3C_2T_x$ MXene as a lattice-match electrons/ions redistributor. ACS Nano **15**(9), 14631-14642 (2021). https://doi.org/10.1021/acsnano.1c04354

[S5] Q. Zhang, J. Luan, X. Huang, L. Zhu, Y. Tang et al., Simultaneously regulating the ion distribution and electric field to achieve dendrite-free Zn anode. Small **16**(35), 2000929 (2020). https://doi.org/10.1002/smll.202000929

[S6] J.H. Park, M.J. Kwak, C. Hwang, K.N. Kang, N. Liu et al., Self-assembling films of covalent organic frameworks enable long-term, efficient cycling of zinc-ion batteries. Adv. Mater. **33**(34), 2101726 (2021). https://doi.org/10.1002/adma.202101726

[S7] Z. Guo, L. Fan, C. Zhao, A. Chen, N. Liu et al., A dynamic and self-adapting interface coating for stable Zn-metal anodes. Adv. Mater. **34**(2), 2105133 (2022). https://doi.org/10.1002/adma.202105133

[S8] N. Zhang, S. Huang, Z. Yuan, J. Zhu, Z. Zhao et al., Direct self-assembly of MXene on Zn anodes for dendrite-free aqueous zinc-ion batteries. Angew. Chem. **60**(6), 2861-2865 (2021). https://doi.org/10.1002/anie.202012322

[S9] Y. Wang, T. Guo, J. Yin, Z. Tian, Y. Ma et al., Controlled deposition of zinc-metal anodes via selectively polarized ferroelectric polymers. Adv. Mater. **34**(4), 2106937 (2022). https://doi.org/10.1002/adma.202106937
